# FOXP family DNA methylation correlates with immune infiltration and prognostic value in NSCLC

**DOI:** 10.3389/fgene.2022.937069

**Published:** 2022-09-09

**Authors:** Dong-Mei Hu, Wen-Di Zhang, Zhuang-E Shi, Meng-Yu Zhang, Rui Li, Qing-Xiang Wang, Xiu-Li Ji, Yi-Qing Qu

**Affiliations:** ^1^ Shandong Key Laboratory of Infectious Respiratory Diseases, Department of Pulmonary and Critical Care Medicine, Qilu Hospital of Shandong University, Jinan, China; ^2^ Laboratory of Basic Medical Sciences, Qilu Hospital of Shandong University, Jinan, China; ^3^ Department of Pulmonary Disease, Jinan Traditional Chinese Medicine Hospital, Jinan, China

**Keywords:** non-small cell lung cancer, FOXP family, prognostic value, immune infiltration, DNA methylation

## Abstract

**Background:** Forkhead box P (FOXP) family was introduced as a double-edged sword in tumorigenesis and influenced immunotherapy response by modulating host immunity. This study aimed to summarize the involvement of the FOXP family in non-small cell lung cancer (NSCLC).

**Methods:** The UALCAN, Gene Expression Profiling Interactive Analysis (GEPIA), and Reverse transcription-quantitative polymerase chain reaction (RT‒qPCR) were used to analyse the expression levels of the FOXP family in NSCLC. The prognostic impact was evaluated using Kaplan-Meier Plotter. MethSurv, UALCAN, and cBioPortal were applied to analyse the DNA methylation and mutation status of the FOXP family respectively. COEXPEDIA, STRING, and GeneMANIA were used to explore the interaction mechanism. Finally, TISIDB was used to investigate all of the immune-related characteristics regulated by the FOXP family.

**Results:** The expression levels of FOXP1/3/4 were dysregulated in NSCLC tissues than that in normal tissues. Groups with low expression levels of FOXP1/4 and high expression levels of FOXP2/3 were associated with poor prognosis in NSCLC. The transcriptional levels of FOXP2/3/4 were correlated with DNA methylation in NSCLC. FOXP1/3/4 DNA methylation were correlated with prognosis. Pathway enrichment analysis indicated the FOXP family was mainly related to immune-related pathways. After DNA methylation, the correlations between FOXP family and immune factors were opposite to that before alteration in NSCLC.

**Conclusion:** This study elucidated FOXP family could serve as vital diagnostic and prognostic biomarkers in NSCLC. Our study highlighted novel potential functions of FOXP family DNA methylation in regulation of immune-related signatures in NSCLC.

## Introduction

Lung cancer accounts for a large proportion of malignant tumours in the world, of which non-small cell lung cancer (NSCLC) accounts for approximately 85% (Hirsch et al., 2017). According to diverse histological subtypes, NSCLC can be divided into lung adenocarcinoma (LUAD) and lung squamous cell carcinoma (LUSC) (Ruiz-Cordero and Devine, 2020). At present, surgery, cisplatin-based therapy, stereotactic body radiation therapy, definitive concurrent chemotherapy, and radiation therapy have significantly reduced the risk of death in NSCLC. However, these treatments are only suitable for a very small proportion of NSCLC patients. Meanwhile, according to data from recent years, the long-term survival rate of NSCLC patients is still very poor (Evison and AstraZeneca, 2020). Most recently, immune checkpoint inhibitors (ICIs), including inhibitors of the programmed cell death receptor 1 (PD-1) axis, have apparently altered the NSCLC management landscape (Camidge et al., 2019). However, effective biomarkers for guiding NSCLC patients to use ICI drugs are still lacking (Zhang et al., 2021). Therefore, investigating the molecular mechanisms that drive NSCLC initiation and progression, searching for more sensitive biomarkers, and identifying biomarkers for ICI efficacy are the current research hotspots. The forkhead box P (FOXP) family consists of four members, including FOXP1, FOXP2, FOXP3, and FOXP4 (Kim et al., 2019). The FOXP family is responsible for the occurrence of many tumours. For example, FOXP1 is related to the occurrence of drug resistance in patients with ovarian cancer during treatment (Hu et al., 2020). FOXP1 also has a function in the occurrence of cancer cachexia that causes weakness (Neyroud et al., 2021). FOXP2 promotes tumour progression in triple-negative breast cancer through the mechanisms of targeting specific molecules (Wu et al., 2018). FOXP3 is involved in the regulation of autophagy-related proteins in gastric cancer (Li et al., 2020a). Overexpression of FOXP4 is closely implicated in the malignant prognosis of breast cancer by promoting the biological process of EMT (Ma and Zhang, 2019). Therefore, we know that the FOXP family plays a role in tumour suppressor genes and oncogenes in tumours (Kim et al., 2019). However, the roles of the FOXP family in the effect and mechanism of immune infiltration have not yet been determined. In this article, we comprehensively analysed FOXP family mRNA expression/DNA methylation signatures, mutations, functional pathways of coexpression networks, survival value, epigenetic alterations, and relationships with immune-related factors. Furthermore, we performed real-time quantitative PCR (RT‒qPCR) to detect the expression levels of the FOXP family.

## Materials and methods

### UALCAN

UALCAN (http://ualcan.path.uab.edu/), an online website was used to compare the difference in the mRNA expression levels of the FOXP family between NSCLC tissues and normal tissues obtained from The Cancer Genome Atlas (TCGA) (Chandrashekar et al., 2017). Then, we explored the changes in FOXP family expression levels in different pathological stages with this tools. In addition, we used UALCAN to analyse the effect of DNA methylation on the translational levels of the FOXP family.

### Gene expression profiling interactive analysis

GEPIA (http://gepia.cancer-pku.cn) was used to analyse the mRNA levels of the FOXP family in NSCLC tissues compared to normal tissues using the open public data from TCGA (Tang et al., 2017). Under the condition of selecting the corresponding cancer species, the website can automatically output the corresponding scatter diagrams, bar charts, and box plots according to the input gene name.

### Kaplan–Meier plotter

Kaplan‒Meier plotter (http://kmplot.com/analysis/) provided data and algorithms for analysing the prognostic significance of patients with expression imbalances of the FOXP family (Peng et al., 2017). All the patients were divided into two groups according to the median expression levels of FOXP family genes to measure the difference in survival time between the above two groups. Kaplan‒Meier curves were plotted to explore the overall survival (OS) analysis by the log-rank test. *p* values < 0.05 were defined as statistically significant.

### MethSurv database

The MethSurv database (https://biit.cs.ut.ee/methsurv/) was used to perform survival analysis of DNA methylation of the FOXP family in NSCLC by selecting a specific gene name and cancer type using the TCGA dataset. The “Region-based analysis” module was used by choosing “LUAD TCGA March 2017” and “LUSC TCGA March 2017”.

### R/Bioconductor package

We visualized the Gene Ontology (GO) and Kyoto Encyclopedia of Genes and Genomes (KEGG) pathway analysis results of coexpressed genes of the FOXP family using R/Bioconductor packages (“BiocManager,” “DOSE,” “cluster Profiler,” “org.Hs.eg.db,” “enrichplot” and “ggplot2”) which were downloaded from Bioconductor (http://www.bioconductor.org/packages/release/bioc/html/). The enrichment analysis results with a *p* value < 0.05 were demonstrated to have great significance.

### TISIDB

TISIDB (http://cis.hku.hk/TISIDB/index.php) was applied to infer the relative abundance of immune-related characteristics of 28 tumour-infiltrating lymphocyte (TIL) types, immunomodulators, chemokines, and receptors regulated by the FOXP family in NSCLC tissues. On the foundation of the mRNA expression of the FOXP family profiles, gene set variation analysis (GSVA) examined which types of immune-related characteristics were regulated by the current genes with epigenetic alterations (copy number alteration and DNA methylation). In addition, TISIDB provided data on the degree of infiltration of immune-related characteristics in NSCLC tissues to infer the regulatory effect of the FOXP family. Finally, TISIDB was applied to explore the expression of the FOXP family in different immune subtypes (Ru et al., 2019).

### Cancer single-cell state atlas

Cancer single-cell state atlas (CancerSEA) (http://biocc.hrbmu.edu.cn/CancerSEA/) provided datasets that was applied to assess the functional roles of the FOXP family in NSCLC. The CancerSEA supported the evaluation of 14 functional states at the single-cell level using public datasets including epithelial-mesenchymal transition (EMT), DNA damage, and so on.

### COEXPEDIA

COEXPEDIA (https://www.coexpedia.org) is an online database. The corresponding predicted target genes were obtained according to the coexpression trend of consistency and the common pathways involved in the regulation of the occurrence and development of disease. COEXPEDIA offered a network reflecting clear interactions between the members of the FOXP family and the corresponding coexpressed genes.

### cBioPortal

cBioPortal (https://www.cbioportal.org/) was used to ascertain the consequence of alteration frequency and mutation type of the FOXP family in NSCLC (Gao et al., 2013). cBioPortal precisely presented the details of all forms of mRNA dysregulation, gene amplification, and deep deletion with the FOXP family in NSCLC patients by the OncoPrint module.

### STRING

STRING (https://string-db.org) was used to construct a protein‒protein interaction (PPI) network for the retrieval of interacting genes (Szklarczyk et al., 2017). In this article, STRING was used to examine the interactions among the FOXP family and determine the hub regulatory genes. The genes not only required a minimum interaction score ≥ 0.4, but were also imported into Cytoscape (version 3.7.2) with the cytoHubba app to screen the modules of the top 10 hub genes.

### GeneMANIA

GeneMANIA (http://www.genemania.org) administers data on protein and genetic interactions, pathways, and coexpression to predict gene clusters with similar functions (Warde-Farley et al., 2010). This site relies on credible evidence sources of literature to forecast functionally identical genes of the FOXP family to clarify the interaction mechanism of the FOXP family.

### Cell lines and culture conditions and reverse transcription-quantitative polymerase chain reaction

A human lung epithelial cell line (BEAS-2B Cell Article: No. CL-0496), LUAD cell lines (A549 Cell Article: No. CL-0016, NCI-H1299 Cell Article: No. CL-0165, and PC9 Cell Article: No. CL-0298), and LUSC cell line (NCI-H226 Cell Article: SNL-388) were purchased from Procell Life Science & Technology Co. Ltd. (Wuhan, China) on 10 December 2021. All cell lines were identified by short tandem repeat (STR) analysis. The human lung epithelial cell line BEAS-2B and the LUAD cell line PC9 were cultured in Dulbecco’s modified Eagle’s medium (DMEM, Gibco). NSCLC cell lines (A549, NCI-H1299, and NCI-H226) were cultured in RPMI 1640. The two types of culture media both contain 10% heat-inactivated foetal bovine serum (FBS). The gas concentration in the incubator was set to 5% CO_2_, and the temperature was set to 37°C. The method of evaluating the gene expression was RT‒qPCR. TRIzol reagent (Invitrogen) was applied to extract total RNA. After the concentration of extracted RNA reached the appropriate standard, we used miRNA reverse transcription and complementary DNA (cDNA) reverse transcription kits to carry out reverse transcription. Then, RT‒qPCR was performed on a Bio-Rad after the corresponding steps were executed according to the manufacturer’s instructions for TB Green Premix Ex Taq II (Takara). Finally, we used the 2^−ΔΔCt^ method to calculate relative mRNA expression. The reference gene was glyceraldehyde 3-phosphate dehydrogenase (GAPDH), and the sequences of the primers for the GAPDH and FOXP family are listed in Supplementary Table S1.

### Statistical analysis

The statistical data were analysed using GraphPad Prism 9.3.1 by Student’s test and ordinary one-way ANOVA to evaluate the differential expression. The statistical data are presented as the mean ± SEM. Kaplan‒Meier Plotter was used to explore the overall survival (OS) analysis by the log-rank test. The prognostic values of single CpGs in DNA methylation analysis were assessed *via* the likelihood-ratio test. *p* values < 0.05 obtained from all the above analyses were defined as statistically significant.

## Results

### The mRNA expression levels of the forkhead box P family in NSCLC

**FIGURE 1:**
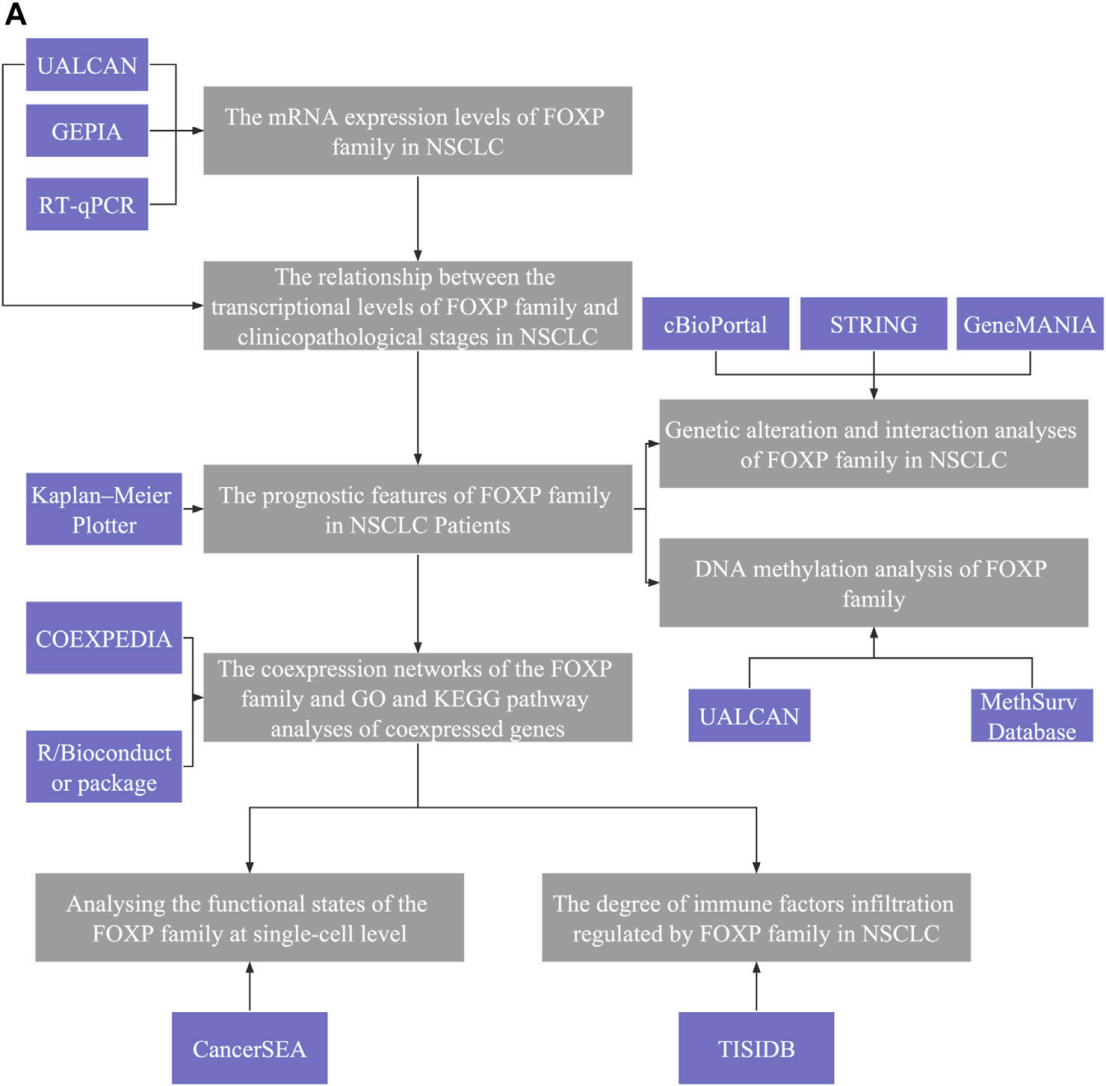
Analysis explanation with a detailed flow diagram of this study. **(A)** The study comprised eight parts: Ⅰ The mRNA expression levels of FOXP family in NSCLC; Ⅱ The relationship between the transcriptional levels of FOXP family and clinicopathological stages in NSCLC; Ⅲ The prognostic features of FOXP family in NSCLC patients; Ⅳ The coexpression networks of the FOXP family and GO and KEGG pathway analyses of coexpressed genes; V Analysing the functional states of the FOXP family at single-cell level; VI The degree of immune factors infiltration regulated by FOXP family in NSCLC; VII Genetic alteration and interaction analyses of FOXP family in NSCLC; VIII DNA methylation analysis of FOXP family.

**FIGURE 2:**
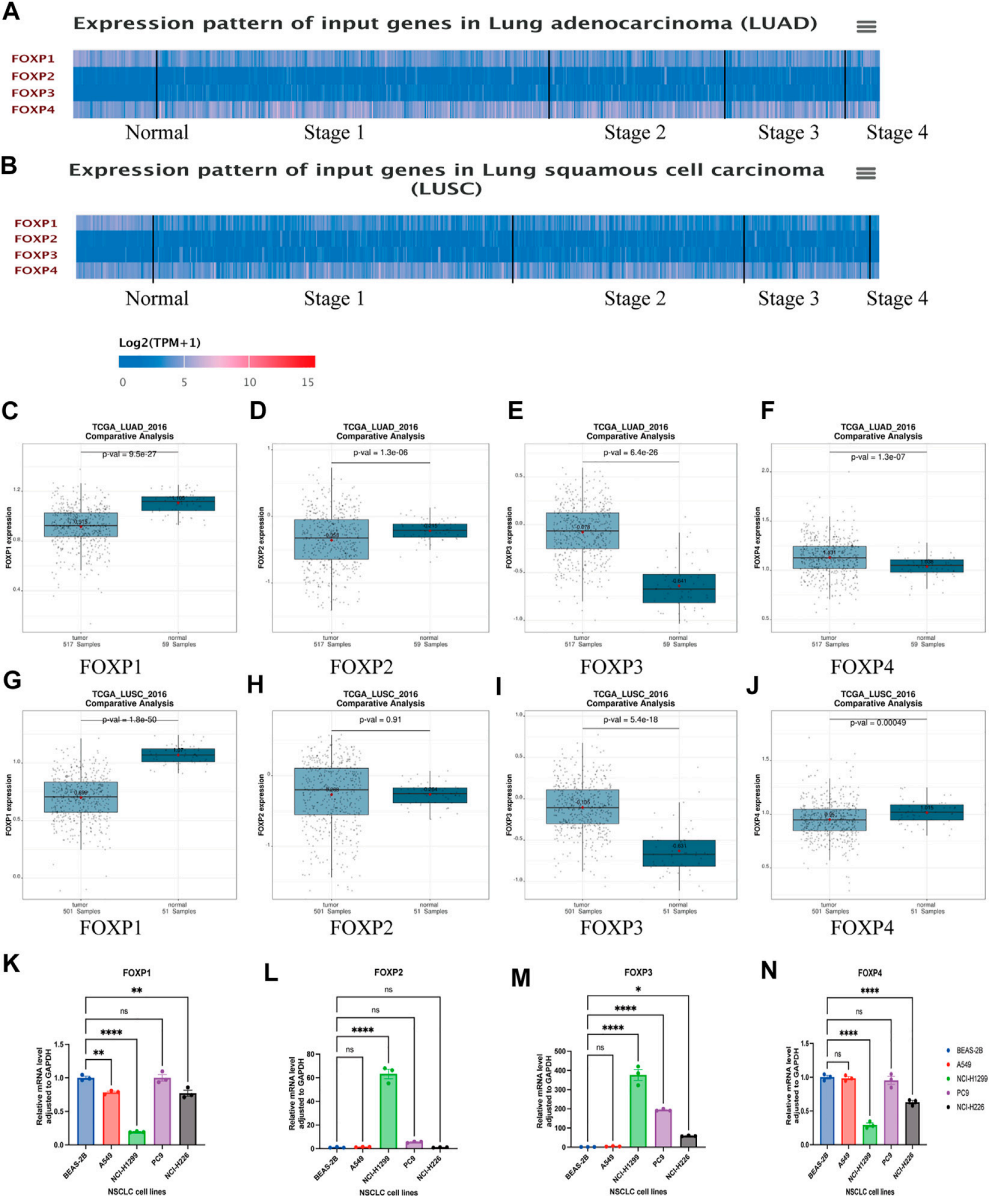
The differential expression of FOXP family in NSCLC. **(A)** The heatmap represented the transcriptional levels of FOXP family in patients with LUAD compared with normal samples using UALCAN. **(B)** The heatmap represented the transcriptional levels of FOXP family in patients with LUSC compared with normal samples using UALCAN. **(C–J)** The compare the mRNA expression of FOXP1/2/3/4 between LUAD/LUSC and normal tissue samples by using GEPIA dataset; The box plot showed the relative expression levels of family in normal tissue and NSCLC tissue. *p* < 0.05 was defined as statistically significant. **(K–N)** The mRNA levels of FOXP family between LUAD cell lines (A549, NCI-H1299, and PC9)/LUSC cell line (NCI-H226) and normal human lung epithelial cell line (BEAS-2B) by RT-qPCR. (Legend: ***p ≤ 0.001; **p ≤ 0.01; *p ≤ 0.05; ns. *p* > 0.05; LUAD, Lung adenocarcinoma; LUSC, Squamous cell carcinoma of lung; FOXP, Forkhead box P; RT-qPCR, Reverse transcription-quantitative polymerase chain reaction).

A flowchart was created to illustrate our study (Figure 1A). UALCAN was used to compare the difference in the mRNA expression levels of the FOXP family between normal samples and NSCLC samples. The summary of the transcriptional levels of the FOXP family is shown in the form of heatmaps (Figures 2A,B). Moreover, the GEPIA database was applied to verify the expression of the FOXP family between NSCLC tissues and normal tissues (Figures 2C–J). Compared to normal tissues, there were lower expression levels of FOXP1 in LUAD and LUSC, a lower expression level of FOXP2 in LUAD, higher expression levels of FOXP3 in LUAD and LUSC, a higher expression level of FOXP4 in LUAD, and a lower expression level of FOXP4 in LUSC. In addition, the expression level of FOXP2 was not significantly different in LUSC. We examined the mRNA expression levels of the FOXP family in cell lines (BEAS-2B, A549, NCI-H1299, PC9, and NCI-H226) (Figures 2K–N). The outcomes of RT-qPCR showed that the mRNA expression levels of FOXP1, FOXP3, FOXP4 did have statistical differences between LUSC cell line (NCI-H226) and normal human lung epithelial cell line (BEAS-2B). However, when tested individually to verify the differential expression levels of FOXP family between LUAD cell lines and normal control, we found that only two members (FOXP1 and FOXP3) were statistically significant between LUAD cell lines (A549, PC9, and NCI-H1299) and normal human lung epithelial cell line (BEAS-2B), which were consistent with analysis of GEPIA database. In order to find the source of this difference, we conducted meta-analysis to explore the expression difference of FOXP family from different database using LUNG CANCER EXPLORER (https://lce.biohpc.swmed.edu/lungcancer/index.php#page-top) database. The results showed that the different expression trends of FOXP2 and FOXP4 objectively existed in LUAD among different data sets. After meta-analysis, it was more likely that the expression of FOXP2 was no statistically significant, and the expression of FOXP4 was upregulated in LUAD patients compared with normal controls (Supplementary Figure S1). Therefore, we concluded that FOXP1 was downregulated, and FOXP3 was upregulated between LUAD patients compared with normal controls, while the expression levels of FOXP2 and FOXP4 in LUAD compared with normal controls need to be verified by more clinical samples. Besides, FOXP1 and FOXP4 were downregulated, FOXP3 was upregulated, and FOXP2 was not statistically significant between LUSC patients with normal controls.

### Relationship between the transcriptional levels of the forkhead box P family and clinicopathological stages in non-small cell lung cancer

**FIGURE 3:**
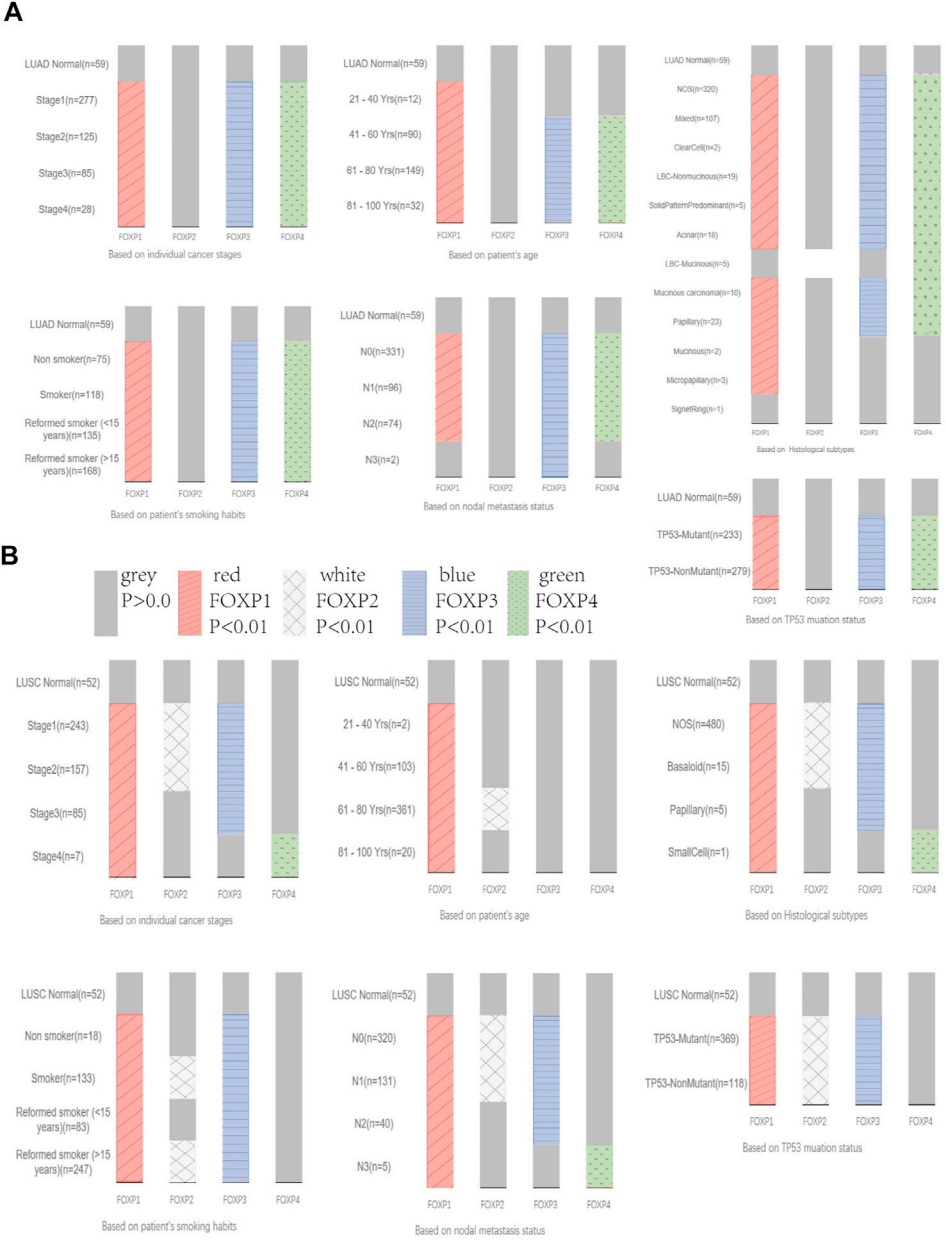
The relationship between the expression levels of FOXP family and clinicopathological stages. **(A)** The Bar graphs showing the expression of FOXP family differences between the clinicopathological stages of LUAD and normal tissues. **(B)** The Bar graphs showing the expression of FOXP family differences between the clinicopathological stages of LUSC and normal tissues.

Next, the inconsistency of the transcriptional expression levels of the FOXP family members among the clinicopathological parameters of NSCLC patients was analysed by UALCAN (Supplementary Figures S2–S5). The clinicopathological parameters included histological subtypes, individual cancer stages, patient age, patient smoking habits, nodal metastasis status, and TP53 mutation status. As shown in the histograms in Figures 3A,B, the transcriptional levels of FOXP1/3/4 were basically markedly correlated with the above six clinicopathological stages in LUAD. However, there was no discernible difference in the relationship between the transcriptional level of FOXP2 and the six clinicopathological stages in LUAD (Figure 3A). The transcriptional levels of FOXP1/2/3 were markedly correlated with the above six clinicopathological stages in LUSC, while the difference in FOXP4 was unremarkable (Figure 3B). In brief, the above results preliminarily suggested that the FOXP family was involved in characteristics that included age factors, inducements, progression, metastasis, and mutation types in NSCLC patients.

### Prognostic features of the forkhead box P family in non-small cell lung cancer patients

**FIGURE 4:**
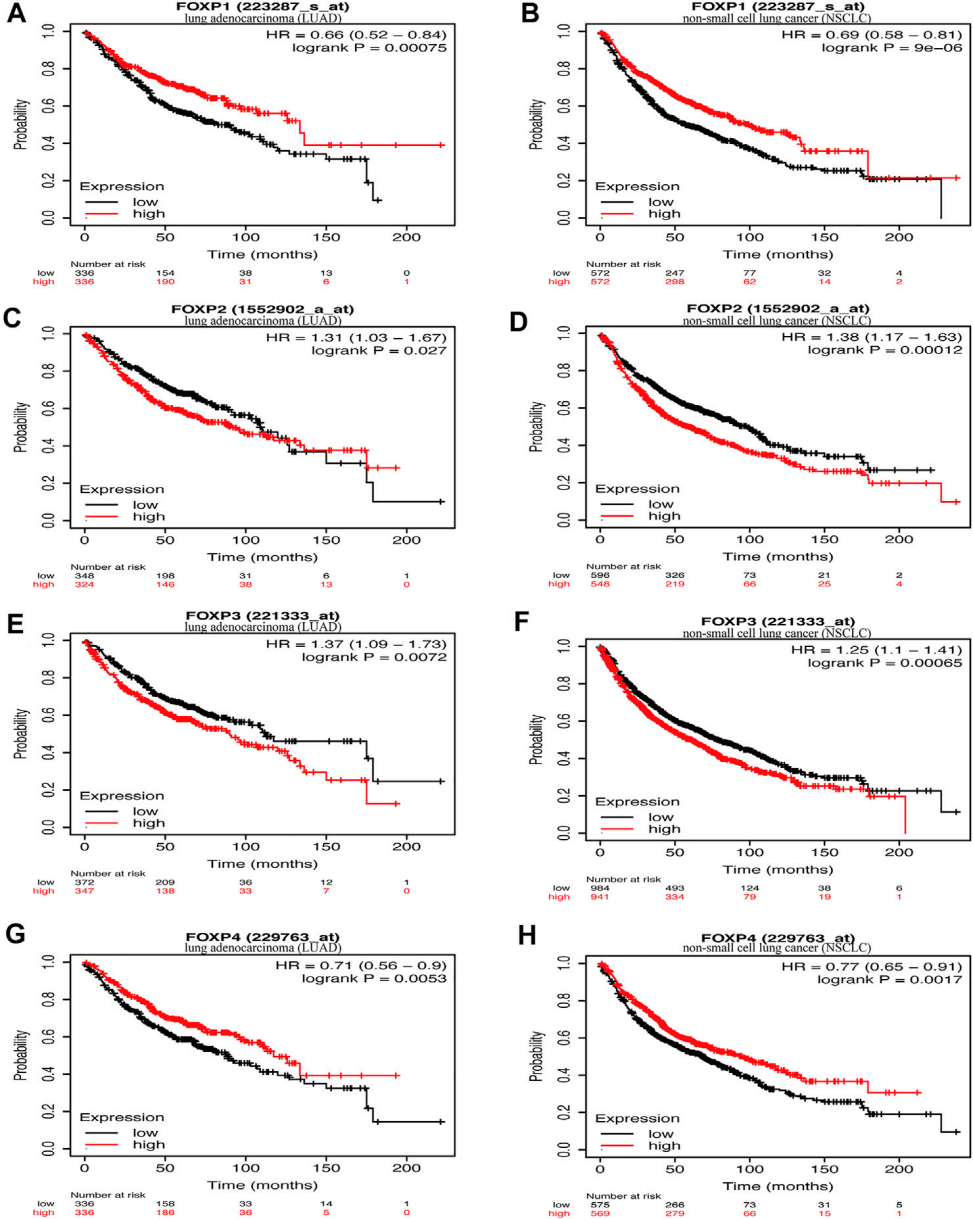
The relationship between the expression of FOXP family and survival. **(A)** The survival curves reflected the relationship between the patients’ overall survival (OS) rate and the corresponding gene expression levels of FOXP1 in LUAD. **(B)** he survival curves reflected the relationship between the patients’ overall survival (OS) rate and the corresponding gene expression levels of FOXP1 in NSCLC. **(C)** The survival curves reflected the relationship between the patients’ overall survival (OS) rate and the corresponding gene expression levels of FOXP2 in LUAD. **(D)** The survival curves reflected the relationship between the patients’ overall survival (OS) rate and the corresponding gene expression levels of FOXP2 in NSCLC. **(E)** The survival curves reflected the relationship between the patients’ overall survival (OS) rate and the corresponding gene expression levels of FOXP3 in LUAD. **(F)** The survival curves reflected the relationship between the patients’ overall survival (OS) rate and the corresponding gene expression levels of FOXP3 in NSCLC. **(G)** The survival curves reflected the relationship between the patients’ overall survival (OS) rate and the corresponding gene expression levels of FOXP4 in LUAD. **(H)** The survival curves reflected the relationship between the patients’ overall survival (OS) rate and the corresponding gene expression levels of FOXP3 in NSCLC.

In this step, Kaplan‒Meier Plotter was used to explore the prognostic value of the FOXP family in NSCLC. Survival curves were generated to present the association between the overall survival (OS) rate of NSCLC patients and the corresponding gene expression levels of the FOXP family. All results are shown in Figures 4A–H; Supplementary Figure S6. Upon stratification according to the median expression level, higher FOXP1 expression was correlated with better prognosis of LUAD (Figure 4A, *n* = 336, hazard ratio (HR) = 0.66, 95% CI 0.52–0.84, log-rank *p* = 0.00075). Higher FOXP1 expression was correlated with better prognosis of NSCLC (Figure 4B, *n* = 572, HR = 0.69, 95% CI 0.58–0.81, log-rank *p* = 9e-06). Lower FOXP2 expression was correlated with better prognosis of LUAD (Figure 4C, *n* = 348, HR = 1.31, 95% CI 1.03–1.67, log-rank *p* = 0.027). Lower FOXP2 expression was correlated with better prognosis of NSCLC (Figure 4D, *n* = 596, HR = 1.38, 95% CI 1.17–1.63, log-rank *p* = 0.00012). Lower FOXP3 expression was correlated with better prognosis of LUAD (Figure 4E, *n* = 372, HR = 1.37, 95% CI 1.09–1.73, log-rank *p* = 0.0072). Lower FOXP3 expression was correlated with better prognosis of NSCLC (Figure 4F, *n* = 984, HR = 1.25, 95% CI 1.1–1.41, log-rank *p* = 0.00065). Higher FOXP4 expression was correlated with better prognosis of LUAD (Figure 4G, *n* = 336, HR = 0.71, 95% CI 0.56–0.9, log-rank *p* = 0.0053). Higher FOXP4 expression was correlated with better prognosis of NSCLC (Figure 4H, *n* = 569, HR = 0.77, 95% CI 0.65–0.91, log-rank *p* = 0.0017). Groups with FOXP1/2/3/4 expression were not associated with prognosis in LUSC patients (Supplementary Figure S6). Overall, groups with low FOXP1/4 and high FOXP2/3 expression were associated with poor prognosis (*p* value < 0.005). Both the high mRNA expression of FOXP1/4 and the low mRNA expression of FOXP2/3 were related to improved prognosis (*p* value < 0.05) in NSCLC patients.

### Coexpression networks of the forkhead box P family and gene ontology and kyoto encyclopedia of genes and genomes pathway analyses of coexpressed genes

**FIGURE 5:**
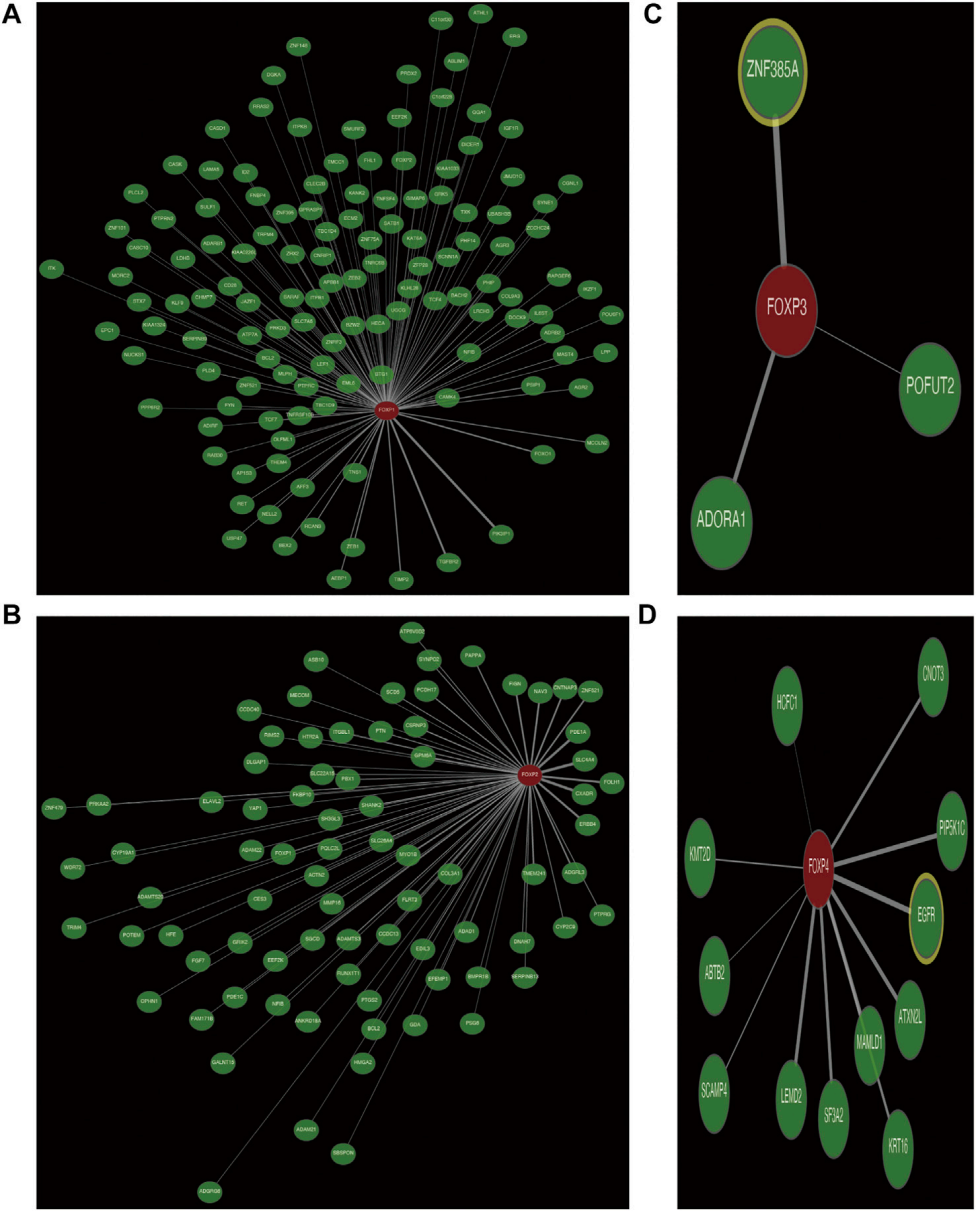
The coexpression network of FOXP family. **(A)** The coexpression network presented the coexpressed genes of FOXP1. **(B)** The coexpression network presented the coexpressed genes of FOXP2. **(C)** The coexpression network presented the coexpressed genes of FOXP3. **(D)** The coexpression network presented the coexpressed genes of FOXP4.

**FIGURE 6:**
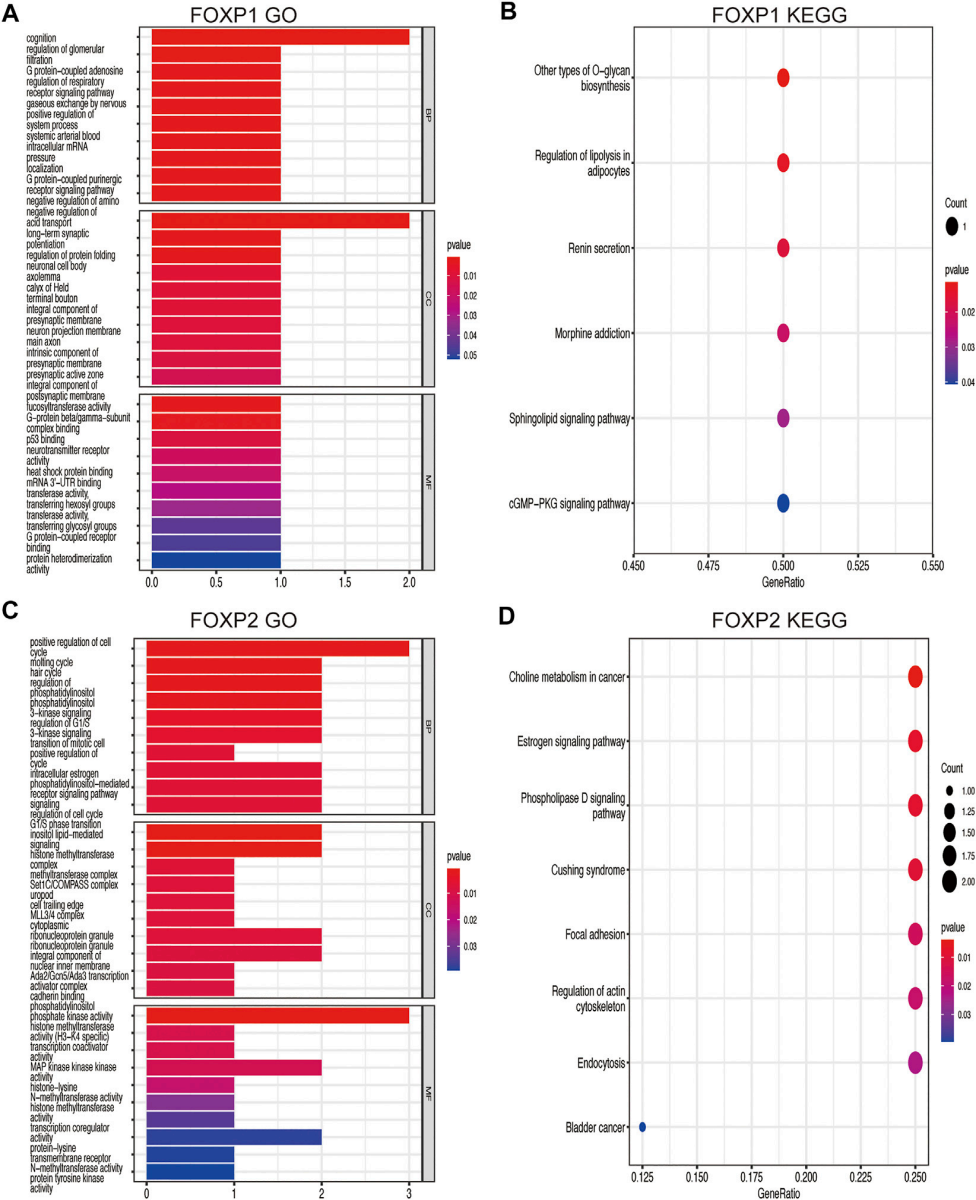
GO functional and KEGG pathway enrichment analyses were performed on the coexpressed genes. **(A)** The GO functional enrichment analysis result on the coexpressed genes of FOXP1 using three annotation systems (BP; CC; MF). **(B)** The KEGG pathway enrichment analysis result on the coexpressed genes of FOXP1. **(C)** The GO functional enrichment analysis result on the coexpressed genes of FOXP2. **(D)** The KEGG pathway enrichment analysis result on the coexpressed genes of FOXP2. (BP, Biological process; MF, Molecular function; CC, Cellular component).

**FIGURE 7:**
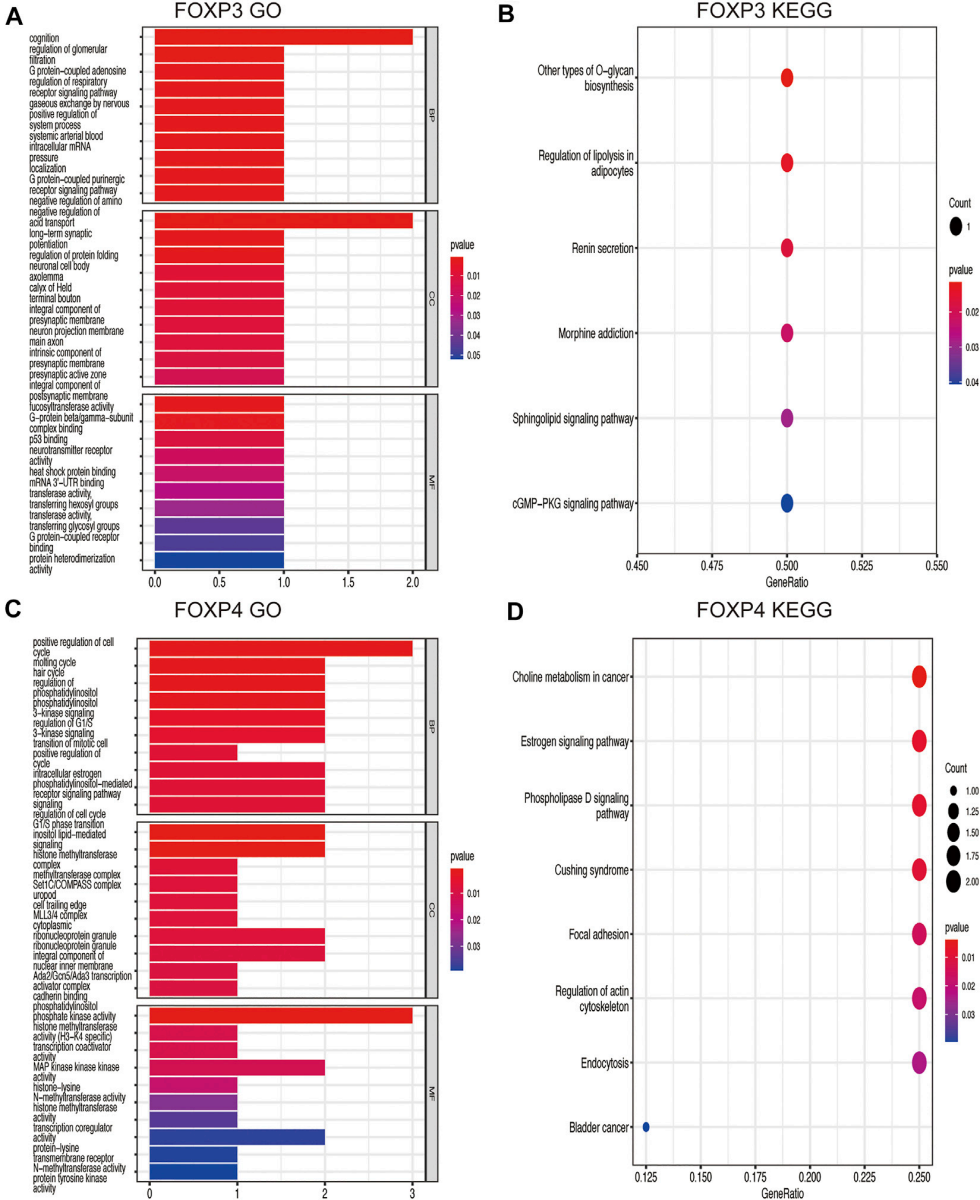
GO functional enrichment analysis and KEGG pathway enrichment analysis are performed on the coexpressed genes. **(A)** The result of GO functional enrichment analysis on the coexpressed genes of FOXP3. **(B)** The result of KEGG pathway enrichment analysis on the coexpressed genes of FOXP3. **(C)** The result of GO functional enrichment analysis on the coexpressed genes of FOXP4. **(D)** The result of KEGG pathway enrichment analysis on the coexpressed genes of FOXP4.

Genes coexpressed with the FOXP family were investigated by the COEXPEDIA website. The coexpression networks of the FOXP family are displayed in Figures 5A–D. The log-likelihood score (LLS score) was used to evaluate the correlations between the FOXP family and its linked genes. The larger the LLS score, the more relevant the coexpression trend of the FOXP family member and its linked genes. The LLS scores of all coexpressed genes are summarized in Supplementary Table S2. GO and KEGG enrichment analyses for coexpressed genes related to the FOXP family were implemented to analyse biological functions and pathways associated with the FOXP family. The biological process (BP), molecular function (MF), and cellular component (CC) of GO enrichment analysis are displayed in Figures 6A,C, 7A,C. In addition, the 20 most relevant KEGG pathways for coexpressed genes are presented in Figures 6B,D, 7B,D. Notably, GO enrichment results showed that the coexpressed genes of the FOXP family mainly acted on the immune process in MF, such as differentiation of immune cells (lymphoid, monocyte, and T cell), fucosyltransferase activity, phosphatidylinositol phosphate kinase activity, transcription coactivator activity, and transcription costimulatory factor regulation. KEGG pathway analysis results showed that coexpressed genes clusters of the FOXP family acted on typical cancer- and immune-related signalling pathways including the T cell receptor, sphingolipid, cGMP-PKG,and phospholipase D signalling pathway. These results strongly implied that the FOXP family was involved in the process of immune regulation in NSCLC.

### Analysing the functional states of the forkhead box P family at the single-cell level

**FIGURE 8:**
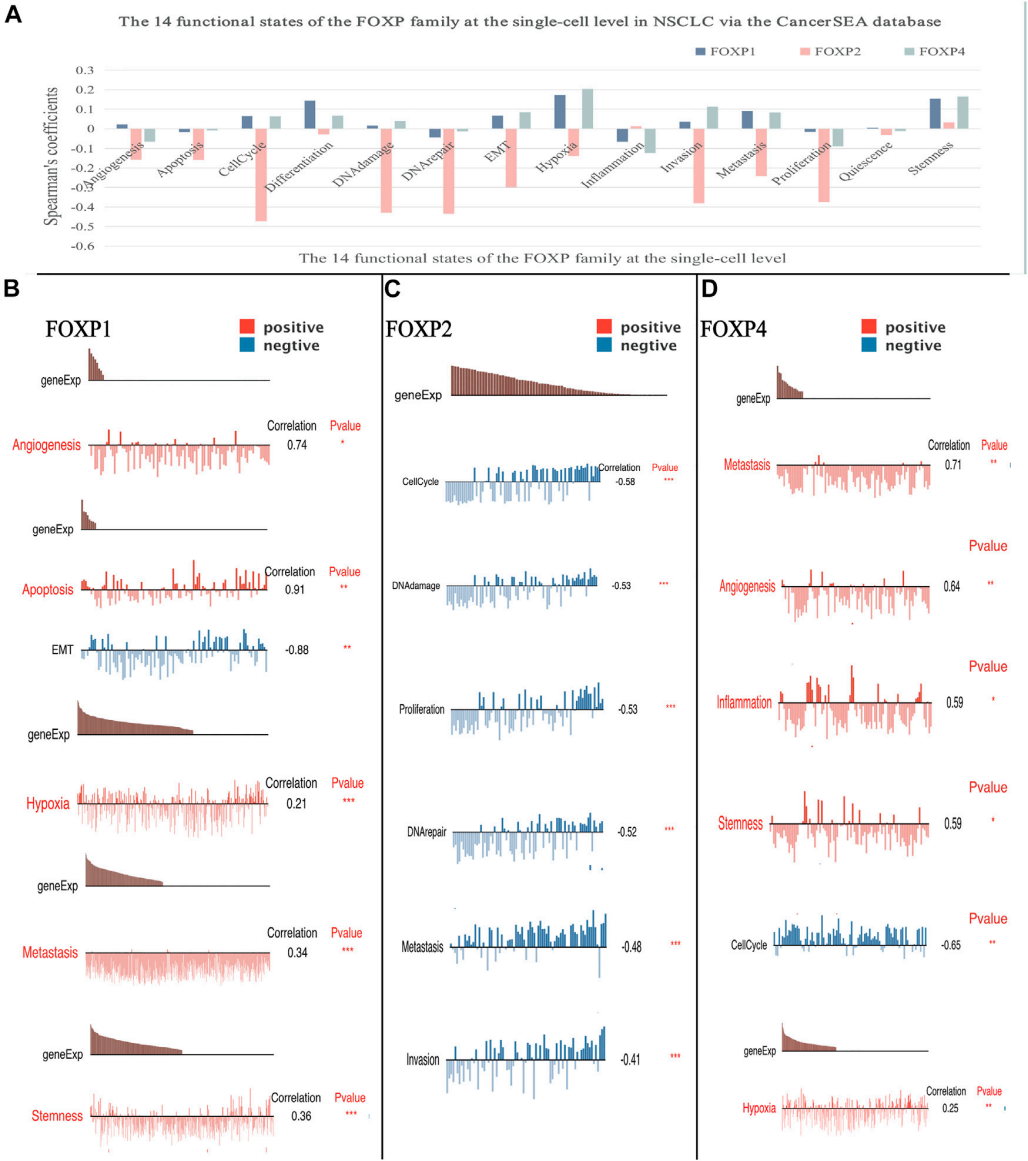
The correlation between the FOXP family and 14 functional states at single-cell level. **(A)** The result of the correlation between expression of the FOXP1/2/4 with functional states (including angiogenesis, apoptosis, invasion, EMT, differentiation, proliferation, DNA damage, metastasis, hypoxia, inflammation, cell cycle, DNA repair, stemness, and quiescence). **(B)** The sample Kim (Exp0068) showed the result of the correlation between the FOXP1 with functional states. **(C)** The sample Kim (Exp0066) showed the result of the correlation between the FOXP2 with functional states. **(D)** The sample Kim (Exp0068) showed the result of the correlation between the FOXP4 with functional states. (EMT, epithelial-mesenchymal transition).

Enrichment analysis results showed that coexpressed gene clusters of the FOXP family acted on several typical cancer pathways. To better understand the relevance and underlying mechanisms of the FOXP family in NSCLC, we investigated the 14 functional states of the FOXP family at the single-cell level *via* the CancerSEA database (Figure 8). The results indicated that FOXP1 was mainly positively correlated with differentiation and hypoxia, FOXP2 was mainly negatively correlated with cell cycle, DNA damage, DNA repair, invasion, metastasis, proliferation, and FOXP4 was mainly positively correlated with hypoxia, invasion, stemness (Figure 8A). Besides, the single-cell analysis result related to FOXP3 were not stated here for the CancerSEA database lacked the FOXP3 data at the single-cell level. we need to supplement this part in the future. In terms of functional relevance in different T cell groups, Kim (Exp0068) showed that FOXP1 had positive correlations with angiogenesis, apoptosis, metastasis, and stemness (Spearman’s coefficients, 0.74, 0.91, 0.34, and 0.36 respectively; *p* value < 0.05) and a negative correlation with EMT (−0.88, *p* value < 0.01) in NSCLC. Kim (Exp0066) showed that FOXP2 had negative correlations with cell cycle, DNA damage, proliferation, DNA repair, metastasis, and invasion(Spearman’s coefficients, −0.53, −0.53, −0.52, −0.48 and −0.41 respectively; *p* value < 0.05) in NSCLC. Kim (Exp0068) reported that high FOXP4 expression was positively correlated with metastasis, angiogenesis, inflammation, stemness and hypoxia, (Spearman’s coefficients, 0.71, 0.64, 0.59, 0.59, and 0.25 respectively; *p* value < 0.05) and negatively associated with cell cycle (Spearman’s coefficients, −0.65, *p* value < 0.01) in NSCLC. These discoveries indicate that the FOXP family may crucially affect the tumour progression of NSCLC.

### The degree of immune factor infiltration regulated by the forkhead box P family in non-small cell lung cancer

**FIGURE 9:**
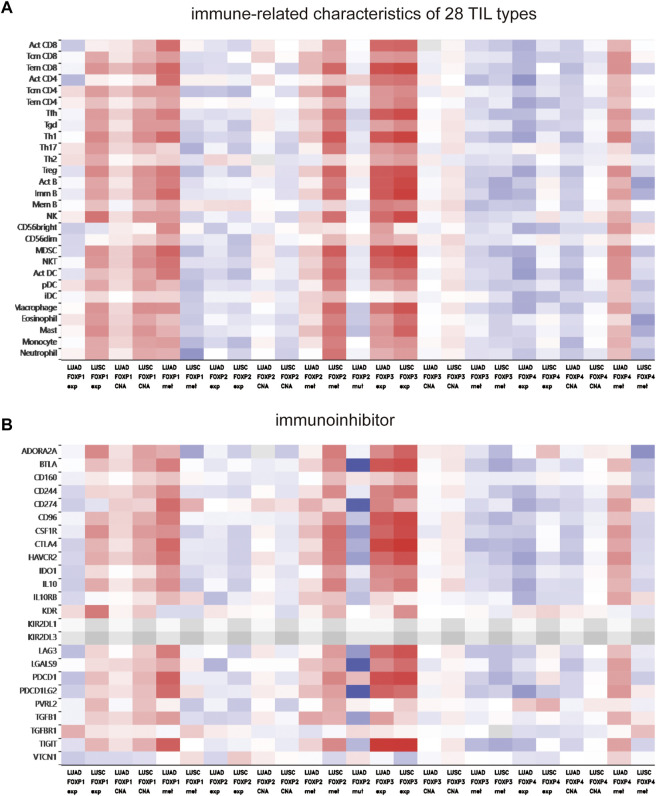
The relationship between the degree of immune factors infiltration in NSCLC and the expression of FOXP family. **(A)** The hotmap presented the correlations between the FOXP family and immune-related characteristics of 28 TIL types. **(B)** The hotmap presents the correlations between the FOXP family and immunoinhibitor.

**FIGURE 10:**
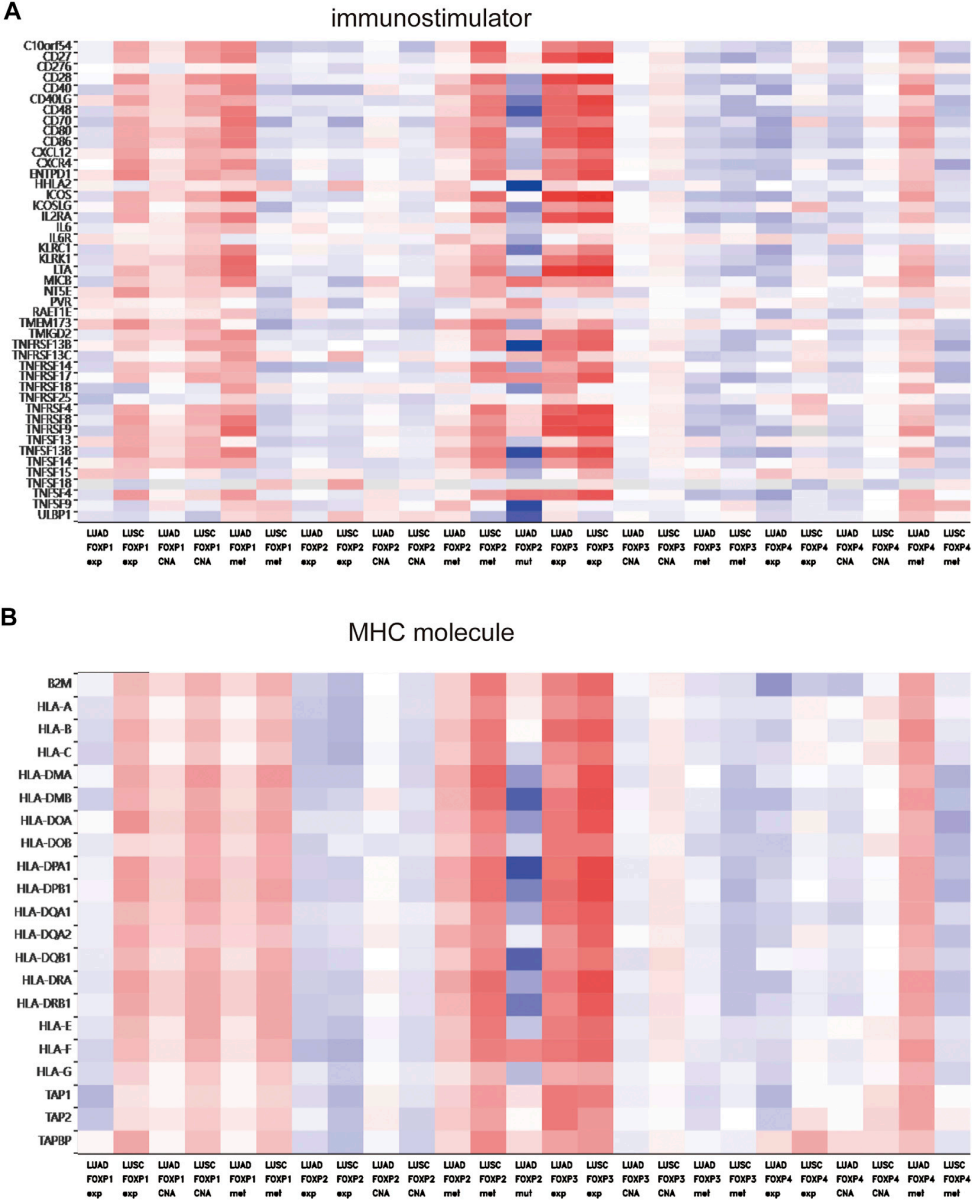
The relationship between the degree of immune factors infiltration in NSCLC and the expression of the FOXP family. **(A)** The heatmap presented the correlations between the FOXP family and immunostimulator. **(B)** The heatmap presented the correlations between the FOXP family and MHC molecule.

**FIGURE 11:**
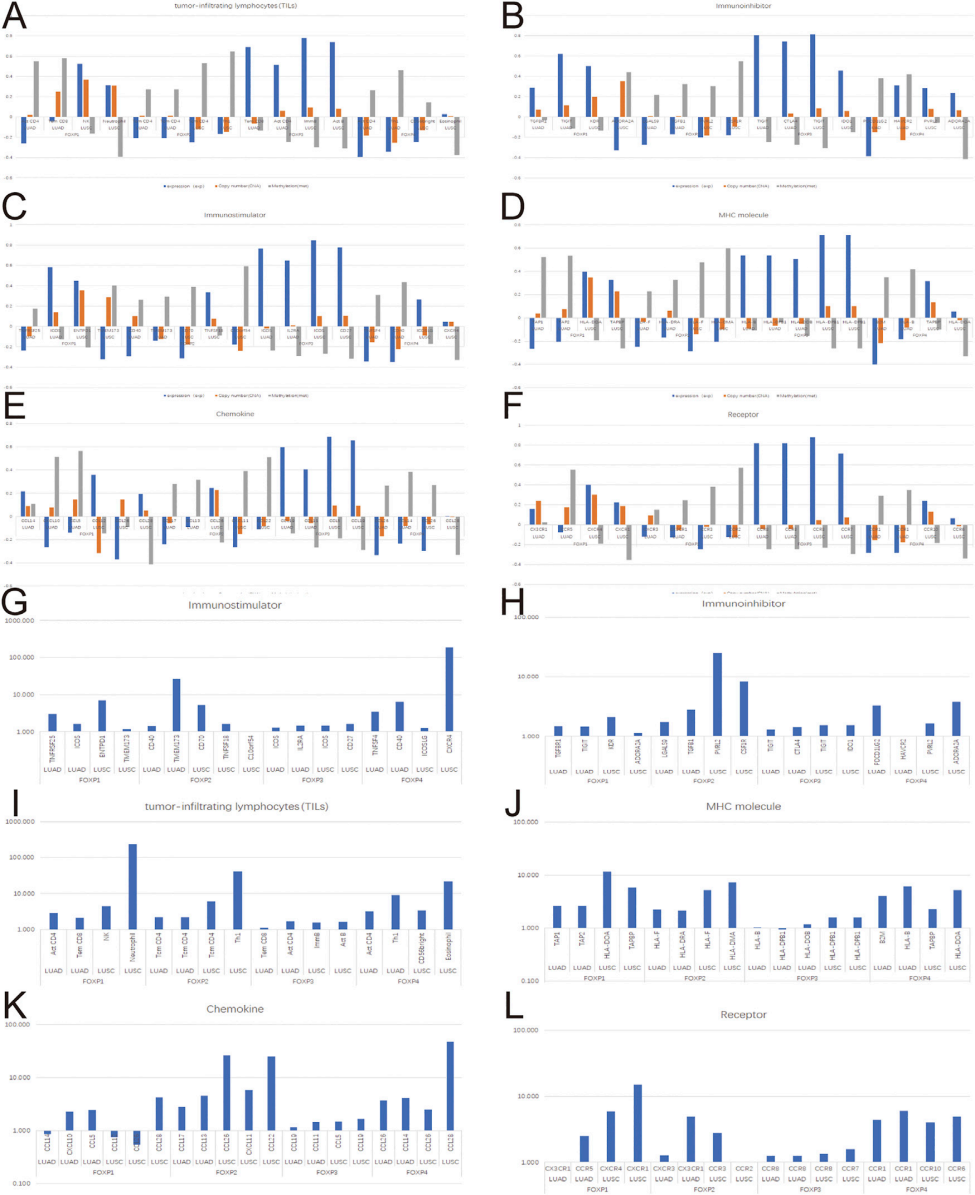
The effects of FOXP family expression, copy number alteration, and DNA methylation on immune factors. **(A–F)** These histograms present the correlation scores of the top two most relevant immune infiltration module and correlation scores modified by copy number alteration and DNA methylation. **(G–L)** These histograms present the fold relationship between copy number alteration and DNA methylation correlation scores of the FOXP family in NSCLC. (CNA, Copy number alteration; MET, DNA Methylation).

**FIGURE 12:**
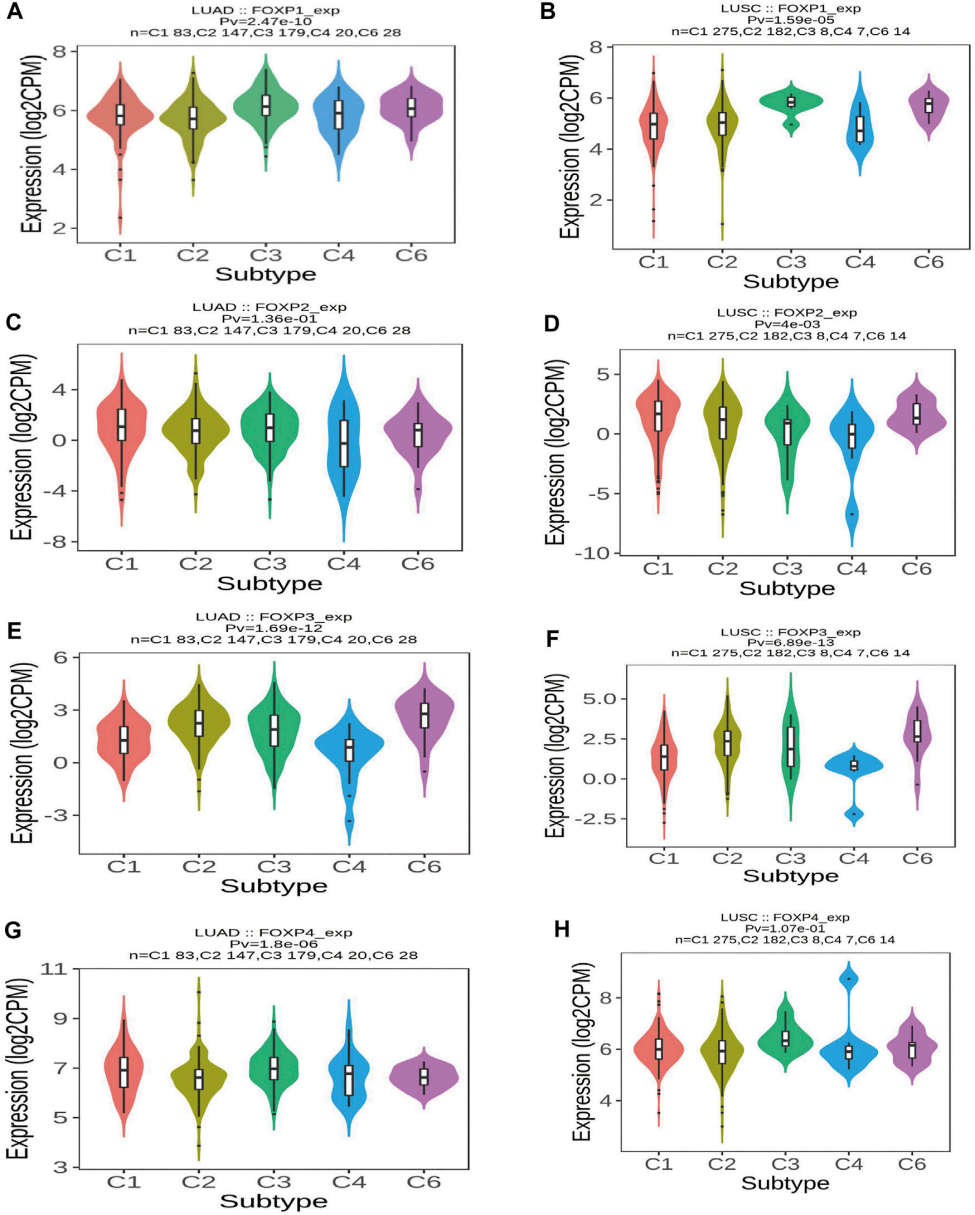
The relationship between immune types and the FOXP family. **(A)** The violins plot showed statistical relationships between FOXP1 and immune types C1–C6 in LUAD. **(B)** The violins plot showed statistical relationships between FOXP1 and immune types C1–C6 in LUSC. **(C)** The violins plot showed statistical relationships between FOXP2 and immune types C1–C6 in LUAD. **(D)** The violins plot showed statistical relationships between FOXP2 and immune types C1–C6 in LUSC. **(E)** The violins plot showed statistical relationships between FOXP3 and immune types C1–C6 in LUAD. **(F)** The violins plot showed statistical relationships between FOXP3 and immune types C1–C6 in LUSC. **(G)** The violins plot showed statistical relationships between FOXP4 and immune types C1–C6 in LUAD. **(H)** The violins plot showed statistical relationships between FOXP4 and immune types C1–C6 in LUSC. [C1 (wound healing); C2 (IFN-gamma dominant); C3 (inflammatory); C4 (lymphocyte depleted); C5 (immunologically quiet); C6 (TGF-b dominant)].

To augment the understanding of the relationship between the FOXP family and immune infiltration, the connection between the FOXP family and various immune signatures, which included the immune-related characteristics of 28 TIL types, immunomodulators (immunoinhibitor, immunostimulator, and MHC molecules), chemokines and receptors, was investigated. All the heatmaps showing the correlation results are presented in Figures 9, 10; Supplementary Figure S7. It was obvious from the heatmaps that the FOXP family was related to immune signatures. To further analyse the relevant mechanisms of the FOXP family in regulating immunity, we selected two modules, the one with the most relevant expression and the other with the most relevant infiltration after copy number alteration and DNA methylation as representatives (the rho of the Spearman correlations test was the highest). When different immune molecules showed upregulation and downregulation trends under the same conditions, two modules were chosen to represent the upregulation and downregulation molecular clusters. The representative immune signatures regulated by FOXP1 were Act CD4 and Tem CD8 in LUAD, NK cells, and neutrophils in LUSC. The infiltration abundances of Act CD4 and Tem CD8 in LUAD tumour tissue were negatively correlated with the expression of FOXP1, and FOXP1 was low in LUAD tissue. That is, the abundances of Act CD4 and Tem CD8 infiltration increased in LUAD tissue. The correlation scores of the two were −0.258 and −0.042, respectively, and there were positive correlations due to copy number alteration and DNA methylation of FOXP1. The representative lymphocytes regulated by FOXP1 in LUSC were NK cells and neutrophils. As we confirmed above, FOXP1 was expressed at low levels in LUSC tissues and was positively correlated with the abundance of NK cells and neutrophil infiltration. The relative rho scores were 0.526 and 0.312, respectively, so NK cells and Neutrophil infiltration were abundant. The degree of decrease in LUSC and was negatively correlated due to the variations in copy number alteration and DNA methylation. By analogy, the regulation of copy number alteration and DNA methylation is shown in the Figures 11A–F. The downregulation of FOXP1 affected the results, including ActCD4, TemCD8, TGFBR1, TIGIT, TNFRSF25, ICOS, TAP1, TAP2, CCL14, CXCL10, CCL5, CX3CR1, CCR5, NK, neutrophils, KDR, ADORA2A, ENTPD1, TMEM1730, HLA-DOA, TAPBP, CCL12, CCL28, CCL26, CXCR4, and CXCR1. The immune infiltration coefficient of FOXP2 in NSCLC tissue was less than those of FOXP1/3/4. The upregulation of FOXP3 mainly affected TemCD8, ActCD4, TIGIT, CTLA4, ICOS, IL2RA, HLA-B, HLA-DPB1, HLA-DOB, CCL19, CCL11, CCR8, ImmB, ActB, TIGIT, IDO1, ICOS, CD27, HLA-DPB1, CCL5, CCL19, CCR8, and CCR7. The downregulation of FOXP4 was mainly associated with ActCD4, Th1, PDCD1LG2, HAVCR-2, TNFSF4, CD40, B2 M, HLA-B, CCL26, CCL14, CCR1, CD56bright, Eosinophil, PVRL2, ADORA2A, ICOSLG, CXCR4, TAPBP, HLA-DOA, CCL26, CCL28, CCR10, and CCR6. We also constructed rate scores to compare the influence of copy number alteration and DNA methylation on the FOXP family (Figures 11G–L). The results presented that both copy number alteration and DNA methylation on the FOXP family play effects on the infiltration correlation results of immune factors in NSCLC, and it was obvious that the changes of immune infiltration correlation after DNA methylation on the FOXP family were significant than those after FOXP family copy number alteration. The multiple influences were different due to different pathological types of NSCLC. Therefore, we could infer that the corresponding conclusion that copy number alteration and DNA methylation regulated the infiltration of corresponding immune factors by the FOXP family. In addition, except for FOXP2 in LUAD and FOXP4 in LUSC, the remaining FOXP family members had significantly different effects on immunophenotyping C1-C6 in NSCLC (Figure 12). Therefore, it was confirmed that the FOXP family participated widely in modulating various immune molecules to affect immune infiltration in NSCLC progression.

### Genetic alteration and interaction analyses of the forkhead box P family in non-small cell lung cancer

**FIGURE 13:**
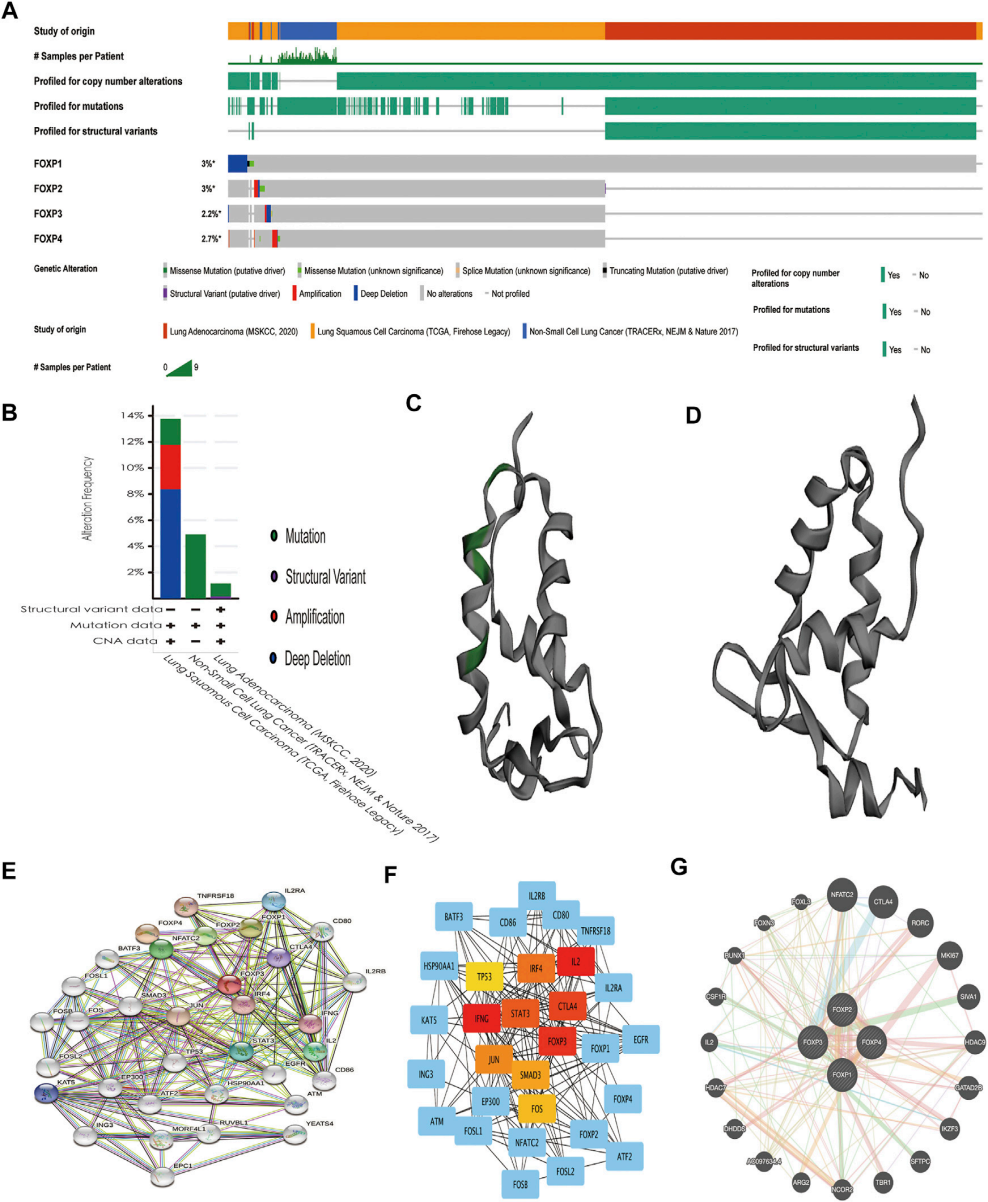
Genetic alteration, and interaction analyses of the FOXP family in NSCLC patients. **(A)** The heatmap showed the respective frequencies of Gene alterations occurring in the sequenced cases by the FOXP family in the data obtained from the OncoPrint schematic of cBioPortal. **(B)** The mutation types of NSCLC in three datasets. **(C)** The chromosomal structure of FOXP1 with domain mutations. **(D)** The chromosomal structure of FOXP2/3/4 without domain mutation. **(E)** The protein-protein interaction PPI network analysis of the FOXP family using STRING. **(F)** The top 10 hub genes were exported by Cytoscape (version 3.7.2) with the cytoHubba app. **(G)** The result of GeneMANIA reveals functionally similar genes of the FOXP family.

Upon analysis of the FOXP family in the OncoPrint module on cBioPortal, the results revealed that gene alterations in FOXP1/2/3/4 occurred in 3%, 3%, 2.2%, and 2.7% of the NSCLC samples, respectively (Figure 13A). The genetic alterations of structural variants, mutations, amplifications, deep deletions, and copy number alterations of the FOXP family all occurred in NSCLC (Figure 13B). The details of all mutations in NSCLC are summarized in Supplement Figure 6. FOXP1 had 15 missense mutations, 3 splice mutations, and one fusion mutation. FOXP2 had one missense mutation and one Fusion mutation. FOXP3 had one missense mutation. FOXP4 had no mutation. Only FOXP1 had domain mutations (Figure 13C), while the remaining FOXP2/3/4 had no domain mutations (Figure 13D). The abovementioned multiple alterations of the FOXP family might partially explain the mechanism of occurrence and progression in NSCLC. In addition, we conducted a PPI network analysis of the FOXP family by STRING to investigate the feasible interactions in their internal and related genes. Multiple nodes (34) and edges (212) are shown in the PPI network (Figure 13E). The STRING results mainly displayed the functions connected with immunity, including regulation of T cell homeostatic proliferation, the activity of T-helper 17 cells, the signalling pathway mediated by interleukin-2, and the adjustment of regulatory T cell differentiation. We further investigated the results of STRING in Cytoscape and then curtained and locked out 10 hub genes (IL2, IFNG, FOXP3, CTLA4, STAT3, IRF4, JUN, SMAD3, FOS, TP53), as shown in Figure 13F. The FOXP family was input to the GeneMANIA website to link genes with similar functions. Functionally similar genes surrounded the outside of the FOXP family in the presentation (Figure 13G). The GeneMANIA results affirmed that the functions of the FOXP family and their related clusters were chiefly related to the differentiation of lymphocytes and T cells and the regulation of leukocytes. The above results support that the FOXP family participates in the immune process of NSCLC under the condition of interaction.

### DNA methylation analysis of the forkhead box P family

**FIGURE 14:**
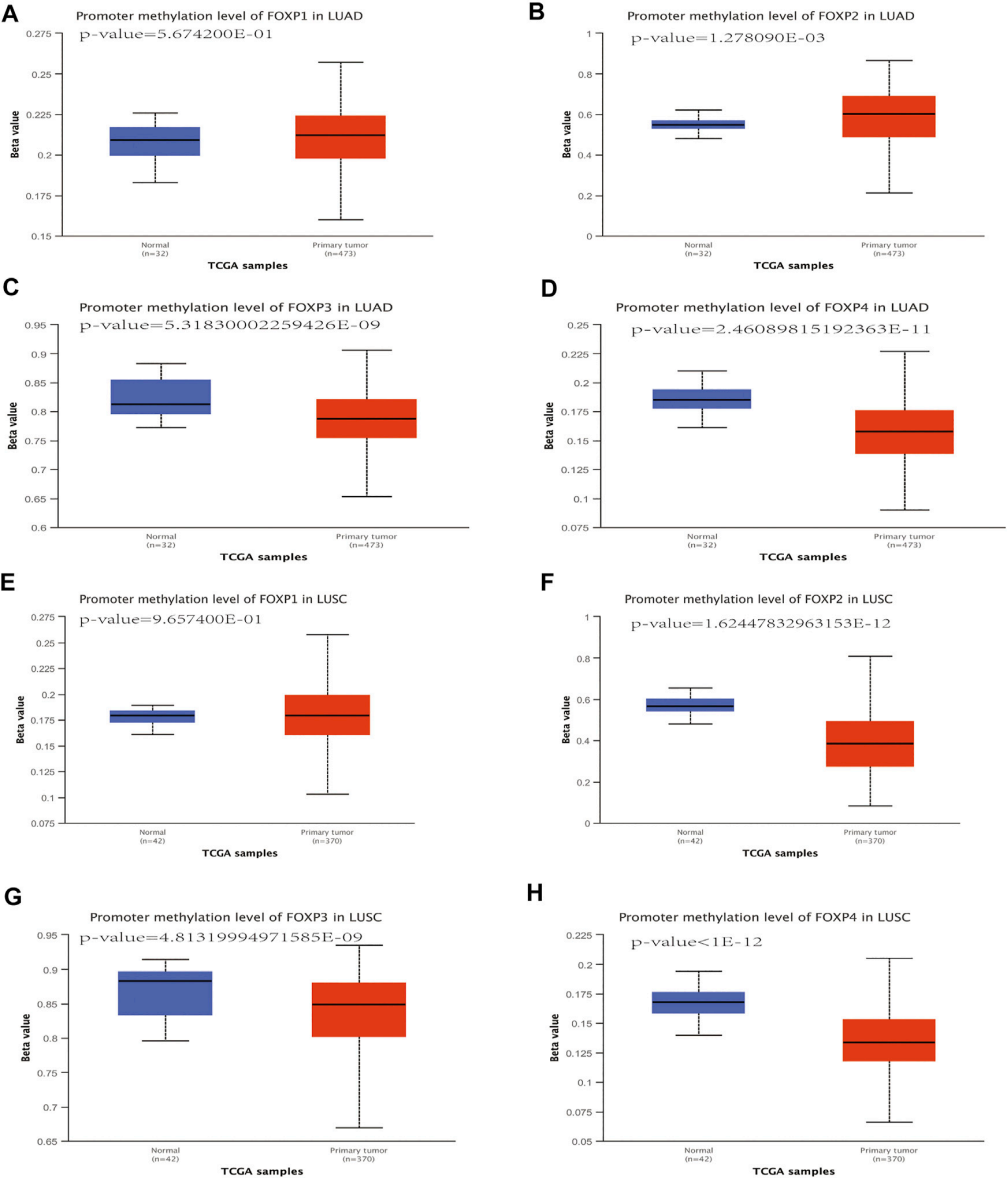
The DNA methylation analysis of FOXP family in NSCLC. **(A–H)** the promoter methylation levels of the FOXP family in LUAD/LUSC compared with normal samples.

In the process of using TISIDB to study the effect of the FOXP family on immune infiltration, we found that the FOXP family significantly changed the correlation degrees between immune signatures after undergoing epigenetic alterations of copy number alteration and DNA methylation. These cBioPortal findings suggested that copy number alteration of the FOXP family played a role in the progression of NSCLC. Therefore, we used DNA methylation as a representative epigenetic alteration to evaluate its effect on the expression levels of the FOXP family and patients prognosis. We first applied the UALCAN database to determine the relationship between DNA methylation and the expression of the FOXP family in NSCLC. The DNA methylation level of FOXP1 was no statistically significant in NSCLC than those in normal samples (Figures 14A,E). The DNA methylation level of FOXP2 was higher in LUAD but lower in LUSC (Figures 14B,F). The DNA methylation levels of FOXP3/4 in NSCLC were lower than those in normal samples (Figures 14C,D,G,H). According to these data, the expression levels of FOXP2/3/4 were significantly associated with DNA methylation in NSCLC. In addition, the results showed that 20 CpGs of FOXP1, 1 CpGs of FOXP3, and 12 CpGs of FOXP4 presented important statistical significance related to prognosis. Moreover, with the occurrence of different CPG sites, FOXP family members’ DNA methylation statuses were related to different prognoses. Specific details of the results including the types of CpG, RefGene groups, relationship to CpG islands, HRs, and *p* values, are listed in Table 1.

**TABLE 1:** The prognostic value of single CpG of FOXP family in NSCLC by MethSurv (*p* < 0.05).

Gene	Tissue	CpG	RefGene group	Relation to CpG island	HR	*p*-value
FOXP1	LUAD	cg00201568	Body	Open_Sea	1.097	0.0026
		cg00707452	TSS1500	S_Shore	0.839	0.0043
		cg01173432	5′UTR	Open_Sea	1.308	0.0066
		cg01186551	5′UTR	Open_Sea	0.923	0.0068
		cg01189917	TSS1500	S_Shore	0.965	0.0076
		cg01232145	Body	Open_Sea	0.746	0.015
		cg01331540	Body	Open_Sea	1.095	0.015
		cg01534217	5′UTR	Island	1.111	0.019
		cg02002523	Body	Open_Sea	0.847	0.02
		cg02220284	Body	Open_Sea	1.318	0.02
		cg02336104	5′UTR	Open_Sea	1.133	0.022
		cg02520804	5′UTR	N_Shore	1.101	0.027
		cg02862354	Body	Island	0.886	0.036
	LUSC	cg22798400	Body	Open_Sea	0.638	0.006
		cg02520804	5′UTR	N_Shore	0.682	0.02
		cg00052246	Body	Open_Sea	1.46	0.034
		cg01173432	5′UTR	Open_Sea	1.564	0.038
		cg00201568	Body	Open_Sea	1.409	0.046
		cg01189917	TSS1500	S_Shore	1.474	0.049
		cg25481160	Body	N_Shelf	0.673	0.05
FOXP2	LUAD	—				
	LUSC	—				
FOXP3	LUAD	—				
	LUSC	cg04920616	TSS200	Open_Sea	0.684	0.032
FOXP4	LUAD	cg12911122	5′UTR	S_Shore	1.759	0.00058
		cg26432961	5′UTR	S_Shore	1.891	0.0035
		cg08696640	5′UTR	S_Shelf	1.558	0.0068
		cg05734456	5′UTR	Island	1.489	0.014
		cg04617914	TSS1500	N_Shore	1.45	0.024
		cg17620505	5′UTR	N_Shelf	1.574	0.028
		cg01508045	5′UTR	Island	1.558	0.029
	LUSC	cg03442064	5′UTR	Island	1.568	0.0057
		cg00806680	Body	N_Shore	0.688	0.022
		cg08727957	TSS1500	Island	0.696	0.026
		cg05140895	TSS200	Island	0.644	0.038

Notes: HR, hazard ratio.

## Discussion

The treatment of NCSLC with immunotherapy including ICIs, has improved the clinical benefits for patients and greatly innovated the traditional chemotherapy regimen (Cogdill et al., 2017). Nonetheless, many patients are still rejected for immunotherapy due to not meeting the inclusion criteria. As a result, research on advanced and effective modulators at immune-related critical points is in full swing. To further understand the molecular regulatory mechanism of the immune system in the management of NSCLC. Our article elaborates on the specific regulatory details of specific immune molecules from the perspective of the FOXP family.

At present, the FOXP family is observed to play negative or positive roles in particular cancers. For example, FOXP1 drives the occurrence of malignant behaviour by dominating the expression level of PKLR in gallbladder cancer (Wang et al., 2019). FOXP2 participates in the process of invasion and metastasis of breast cancer *via* the TGFβ/SMAD pathway (Chen et al., 2018). Aberrant expression of FOXP3 in colorectal cancer is related to immune overdrive in a high-risk subpopulation (Cui et al., 2021). FOXP4 directly acts on LEF-1 and gives impetus to the occurrence of laryngeal squamous cell carcinoma (Shi et al., 2021). At the same time, many recently published works in the literature show that the FOXP family participates in the process of immune system reconstruction of tumour tissue by activating or inhibiting the specific function of immune molecules (Fleskens and van Boxtel, 2014). The FOXP family, as a major contributor, can regulate tumour-associated inflammation and immune responses in tumour progression. For example, FOXP1 inhibits the behaviour of immune activation and the expression of MHC class II in diffuse large B-cell lymphomas (Brown et al., 2016). FOXP3 is defined as a manager to administer the immunosuppressive response of T cells (Klimenko, 2011). FOXP3 directly restrains CD44 breast cancer by participating in the corresponding regulatory role (Zhang et al., 2015). Furthermore, increasing evidence unambiguously confirms that epigenetic alteration plays a role in the process of cancer, and various epigenetic alterations can be used as maker molecules to evaluate the risk of tumour prognosis (Richardson et al., 2018; Zhang and Zhang, 2020). For instance, the regulation of immune cells is closely related to the copy number alteration of TRPV1 in renal cell carcinoma (Zheng et al., 2020). Abnormal DNA methylation impacts gene expression and survival time in breast cancer patients (Gyorffy et al., 2016). Therefore, the underlying mechanism of FOXP family expression/copy number alteration/DNA methylation in the regulation of immune-related signatures was initially elucidated in this paper.

Previous studies have shown that FOXP3 is overexpressed to facilitate the invasion and metastasis of NSCLC (Li et al., 2021). Our results showed there were different expression levels of the FOXP family according to different pathological types in NSCLC compared with normal tissue. UALCAN presented that the expression levels of the FOXP family had significant effects on the clinical parameters, including patient age, smoking habits, histological subtypes, individual cancer stages, nodal metastasis status, and TP53 mutation status. The Kaplan‒Meier Plotter showed that the overexpression levels of FOXP1/4 were involved in the better prognosis, and the overexpression levels of FOXP2/3 were associated with poor prognosis of NSCLC. It may be an option to analyse the mRNA expression levels of the FOXP family members in NSCLC patients to provide powerful markers to define prognosis.

GO and KEGG pathway analyses of coexpressed genes of the FOXP family indicated that the FOXP family possessed roles in activating the Wnt, PI3K/AKT/mTOR, and FOCAD-FAK pathways to regulate tumourigenesis and the progression of relevant immune responses in NSCLC. Combined with previous research, the above typical pathways were associated with NSCLC progression (Heavey et al., 2014; Stewart, 2014; Liu et al., 2020). Our study further clarified the role of the FOXP family in the development of NSCLC. Likewise, CancerSEA showed the functional states of the FOXP family have a necessary relationship with the activity of the cell cycle, differentiation, apoptosis, angiogenesis, invasion, EMT, proliferation, hypoxia, inflammation, and stemness at the single-cell level. The results validated previous evidence that the FOXP family was involved in the progression of a variety of cancers. For example, the FOXP family regulates *β* cell proliferation in concert with NFATC2 (Simonett et al., 2021). Furthermore, FOXP2 targets GRP78 in breast cancer to promote tumour proliferation and metastasis (Wu et al., 2018). In addition, FOXP1 inhibits guidance proteins to promote angiogenesis in cell activity (Grundmann et al., 2013). Taken together, the functional states of the FOXP family accurately revealed that the FOXP family might crucially affect the progression of NSCLC. The results of PPI interaction and GeneMANIA analyses further demonstrated the occurrence of cooperation and interaction between FOXP members. These results implied that FOXP members could function through alliance mechanisms in NSCLC. Our study showed that the FOXP family was prominently dysregulated in NSCLC, and we then carried out the an analysis of genetic alterations. Unsurprisingly, there was the evidence of fusion, mutation, and amplification of the FOXP family in NSCLC. These genetic alterations were undoubtedly involved in the molecular malignant behaviour of NSCLC.

The DNA methylation process of specific genes mediates different biological results of cancer. For example, DNA methylation is related to the occurrence of drug resistance in patients with glioblastoma during treatment (Lu et al., 2020). In addition, the absence of DNA methylation can cause immune evasion in various cancers (Jung et al., 2019). To explore the particular mechanism of the FOXP family in NSCLC, we investigated the connection between the promoter methylation levels and the expression levels of the FOXP family using UALCAN databases. The outcomes showed that the expression levels of FOXP2/3/4 were correlated with their promoter methylation levels in NSCLC. In addition, we analysed the relationship between DNA methylation modification behaviour at different sites of the FOXP family and patient survival time. Significant prognostic values (*p* value < 0.05) were observed for FOXP1/3/4. In short, analysis of FOXP family DNA methylation provides a new approach to the prognosis of NSCLC.

Data from recent years have shown that the combined use of ICIs has improved the survival time of NSCLC patients by blocking the checkpoint inhibition process. Our study presented the correlations between FOXP family expression/copy number alteration/DNA methylation and immune signatures. The results showed that the FOXP family without epigenetic alterations mainly controlled the degrees of infiltration of immune-related factors (Tem CD8, TXNDC5, TAP1, TAP2, CCL5, NK, KDR, ENTPD1, and HLA-DOA) in NSCLC. Previous studies have confirmed that Tem CD8 inhibits tumour growth in mouse models and plays a vital role in cancer immune surveillance and treatment (Wang et al., 2020). TXNDC5 promotes pulmonary fibrosis by augmenting TGFβ signalling through TGFBR1 stabilization (Lee et al., 2020). TAP1 and TAP2 are typical tumour predictors (Gostout et al., 2003; Henle et al., 2017). CCL5, as a receptor antagonist, plays a positive role in the process of tumour progression by attracting macrophages (Van Damme et al., 2004). The activation of NK cells is related to immune dysfunction and a harmful tumour microenvironment (Li et al., 2020b). There is currently a small-molecule tyrosine kinase inhibitor (Moulder, #137) for KDR that is effective for lung cancer (Dai et al., 2019; Song et al., 2020). FOXP3 regulates the expression and infiltration of ENTPD1 to promote the occurrence of tumours (Sun et al., 2010). HLA-DOA has confirmed that the degree of infiltration in the tissue is directly proportional to the degree of inflammation (Okada et al., 2016). After copy number alteration and DNA methylation, our results revealed that the correlations between the FOXP family and immune parameters were opposite to those before alteration in NSCLC. In addition, the influence of DNA methylation was stronger than that of copy number alteration. In addition, due to the different pathological types of NSCLC, the multiples of the influence intensity were also different. Altogether, our results partly showed that FOXP family expression/copy number alteration/DNA methylation regulated the infiltration of corresponding immunity in NSCLC. This paper provided more detailed molecular mechanisms for the development of new immune checkpoints from the perspective of FOXP family.

Our research has many details that need to be further verified. The data required for the content of bioinformatics analysis in this paper are from public databases. Further basic and clinical trials are still required to explore the detailed molecular mechanism of the FOXP family in NSCLC.

## Conclusion

This paper systematically analysed molecular mechanism of FOXP family member regulation, including the expression levels, correlation with clinicopathological stages, DNA methylation levels, epigenetics alterations, prognostic values, relationship with immune regulation and functional analysis based on coexpression in NSCLC. Activation of FOXP family-related pathways could significantly change the patient’s response to tumour immunity. Our article showed that the FOXP family members, as diagnostic and prognostic biomarkers, provide new information for the development of ICI drugs for patients with NSCLC.

## Data Availability

The datasets presented in this study can be found in online repositories. The names of the repository/repositories and accession number(s) can be found in the article/Supplementary Material.

## Supplementary material

SUPPLEMENTARY FIGURE S1 The meta-analysis results of the expression difference of FOXP family from different data sets using LUNG CANCER EXPLORER database. The meta-analysis result of the expression level of FOXP1 from different LUAD/LUSC data sets. **(B,F)** The meta-analysis result of the expression level of FOXP2 from different LUAD/LUSC data sets. **(C,G)** The meta-analysis result of the expression level of FOXP3 from different LUAD and LUSC data sets. **(D,H)** The meta-analysis result of the expression level of FOXP4 from different LUAD and LUSC data sets.

SUPPLEMENTARY FIGURE S2 The relationship between the expression levels of FOXP family and clinicopathological stages. **(A-L)** AuthorAnonymous, The Box plots show the differential expression of FOXP1 between different staged tissues of LUAD/ LUSC and normal tissues.

SUPPLEMENTARY FIGURE S3 The relationship between the expression levels of FOXP family and clinicopathological stages. AuthorAnonymous, **(A-L)** The Box plots show the differential expression of FOXP2 between different staged tissues of LUAD/ LUSC and normal tissues.

SUPPLEMENTARY FIGURE S4 The relationship between the expression levels of FOXP family and clinicopathological stages. AuthorAnonymous,**(A-L)** The Box plots show the differential expression of FOXP3 between different staged tissues of LUAD/ LUSC and normal tissues.

SUPPLEMENTARY FIGURE S5 The relationship between the expression levels of FOXP family and clinicopathological stages. AuthorAnonymous,**(A-L)** The Box plots show the differential expression of FOXP4 between different staged tissues of LUAD/ LUSC and normal tissues.

SUPPLEMENTARY FIGURE S6 The relationship between the expression of FOXP family and survival in LUSC.**(A)** The survival curves reflected the relationship between the patients’ overall survival (OS) rate and the corresponding gene expression levels of FOXP1 in LUSC. **(B)** The survival curves reflected the relationship between the patients’ overall survival (OS) rate and the corresponding gene expression levels of FOXP2 in LUSC. **(C)** The survival curves reflected the relationship between the patients’ overall survival (OS) rate and the corresponding gene expression levels of FOXP3 in LUSC. **(D)** The survival curves reflected the relationship between the patients’ overall survival (OS) rate and the corresponding gene expression levels of FOXP4 in LUSC.

SUPPLEMENTARY FIGURE S7 The relationship between the degree of immune factor infiltration in NSCLC and the expression of FOXP family.**(A)** The hotmap presented the correlations between FOXP family and chemokine. **(B)** The hotmap presented the correlations between FOXP family and receptor.

## Abbreviations

FOXP, Forkhead box P; LUAD, lung adenocarcinoma; LUSC, lung squamous cell carcinoma; NSCLC, non-small cell lung cancer; GEPIA, Gene Expression Profiling Interactive Analysis; GEO, Gene Expression Omnibus; TCGA, The Cancer Genome Atlas; DEG, differentially expressed genes; GTEx, Genotype-Tissue Expression; GAPDH, glyceraldehyde 3-phosphate dehydrogenase; RT-qPCR, Reverse transcription-quantitative polymerase chain reaction; EMT, epithelial-mesenchymal transition; GO, Gene Ontology; CC, cellular component; MF, molecular function; KEGG, Kyoto Encyclopedia of Genes and Genomes; MHC, Major Histocompatibility Complex; OS, The Overall Survival; ICIs, immune checkpoint inhibitors; PD-1, programmed cell death receptor 1; PD-L1, programmed cell death 1 ligand 1; CTLA-4, cytotoxic T-lymphocyte-associated protein 4; PPI, protein-protein interaction; LLS, log-likelihood scores; TILs, tumour-infiltrating lymphocytes; TKI, tyrosine kinase inhibitor; HR, hazard ratio.
