# Supplementary figures and images for: FOXP family DNA methylation correlates with immune infiltration and prognostic value in NSCLC
Source: Front Genet. 2022 Sep 9;13:937069. doi: 10.3389/fgene.2022.937069 (PMC9500381; doi:10.3389/fgene.2022.937069)

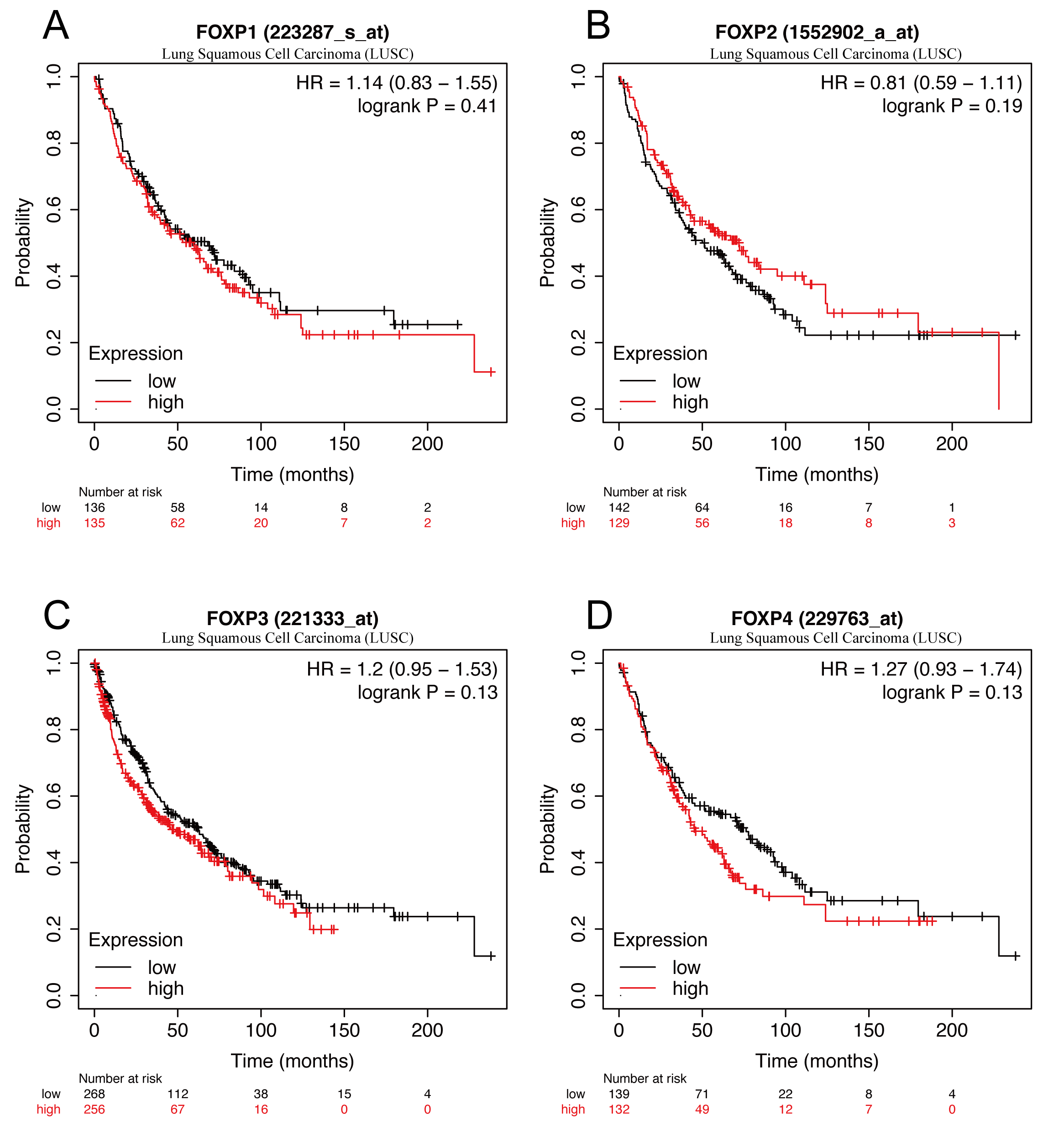

Supplement: Supplementary file 1 [file Image6.tif]

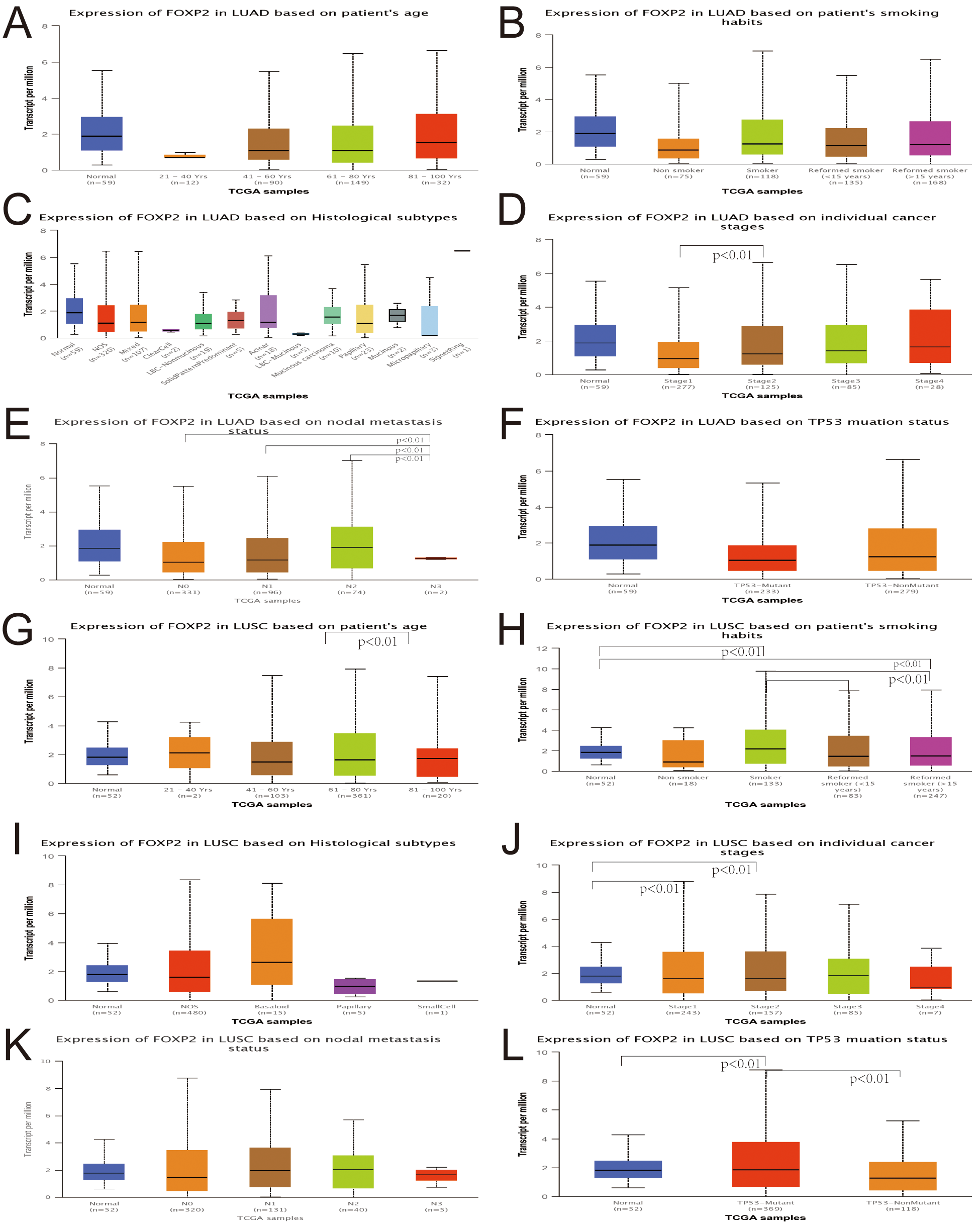

Supplement: Supplementary file 3 [file Image3.tif]

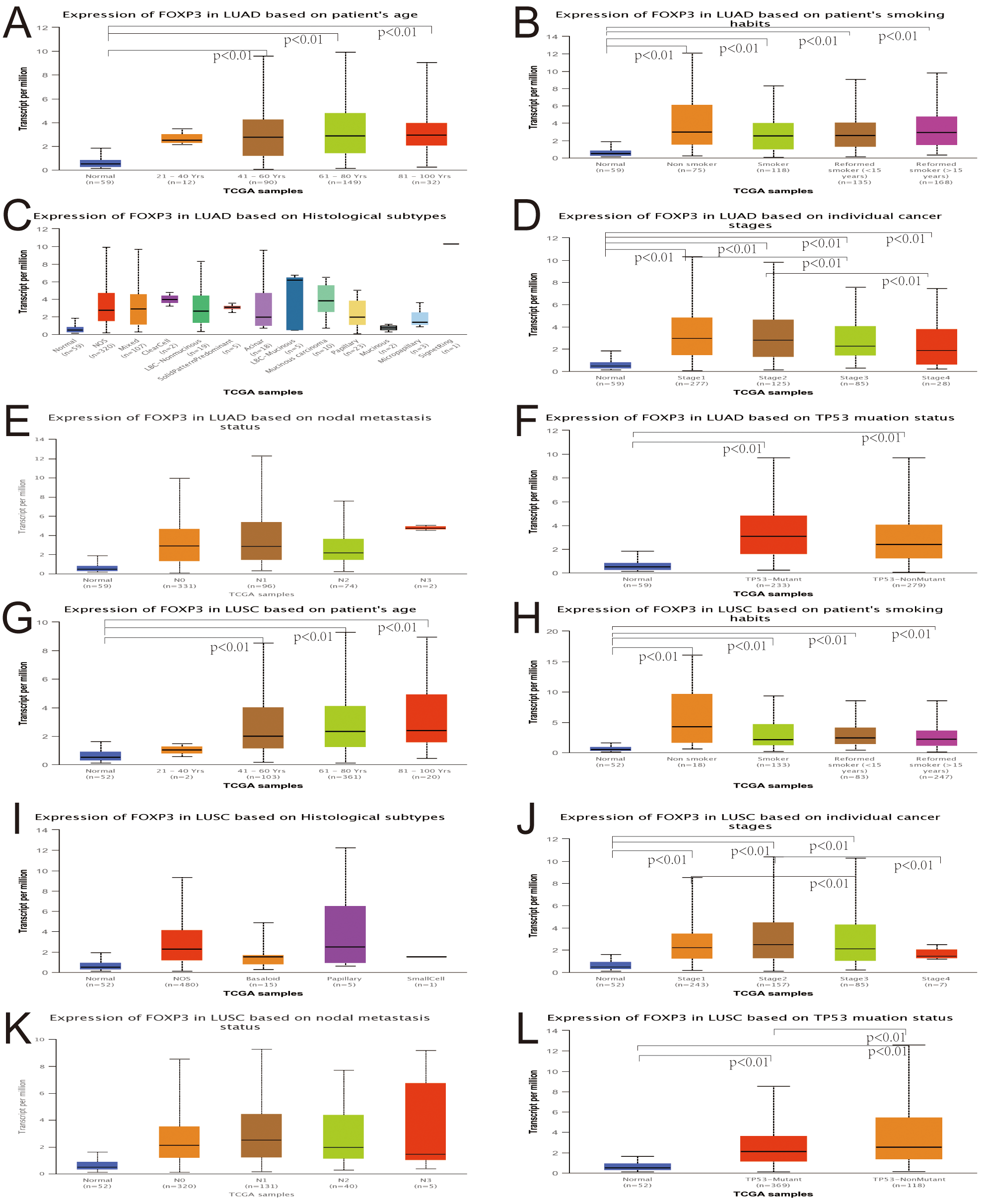

Supplement: Supplementary file 4 [file Image4.tif]

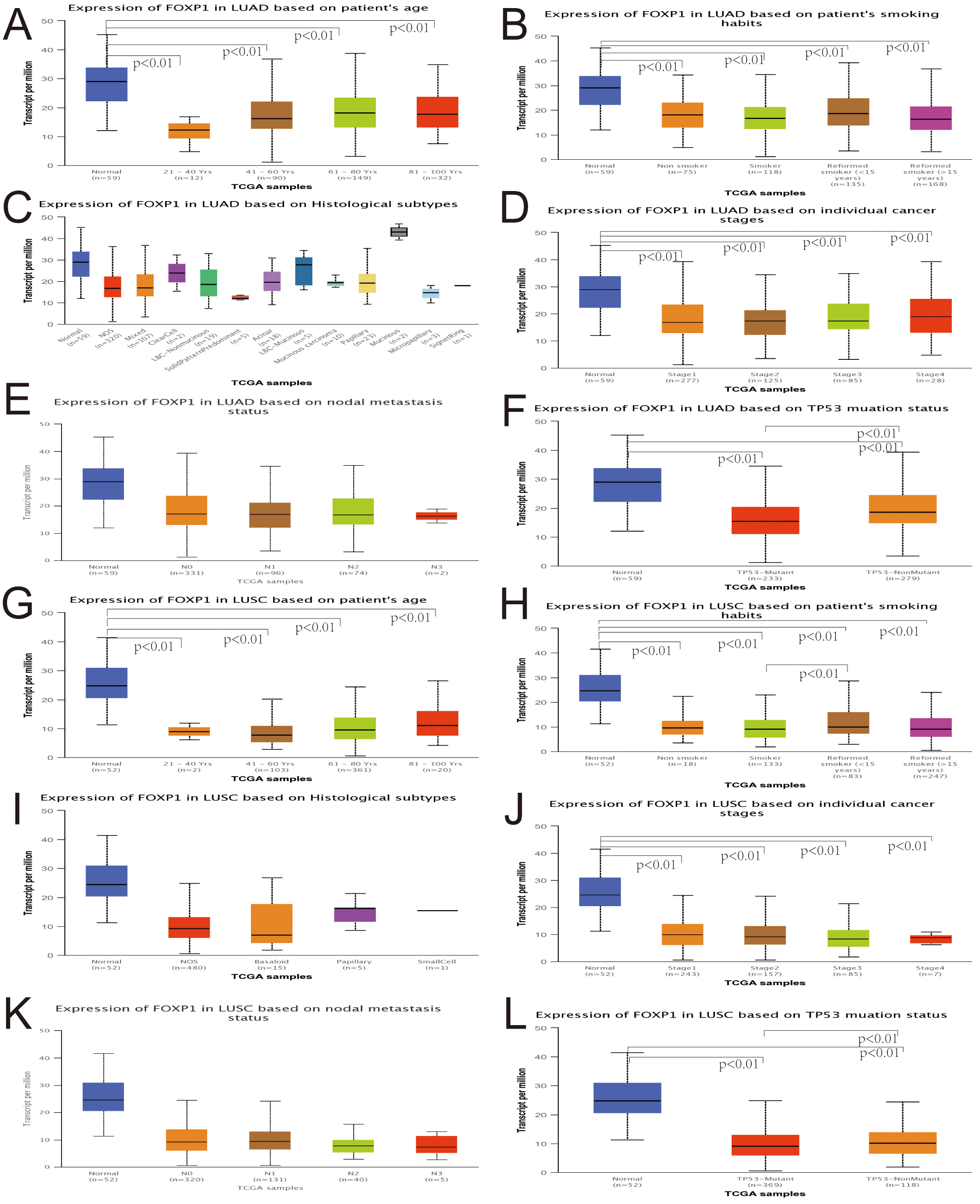

Supplement: Supplementary file 5 [file Image2.tif]

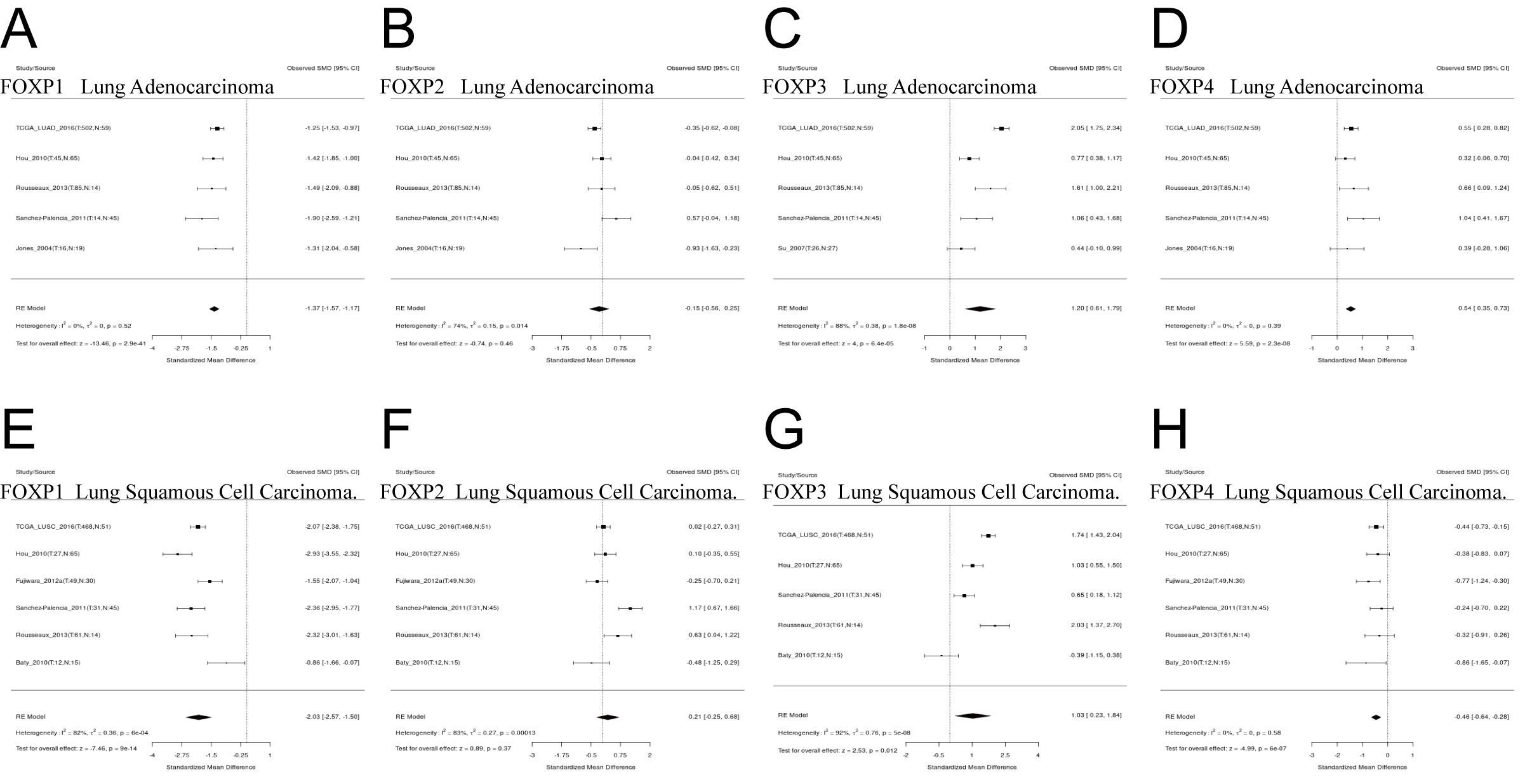

Supplement: Supplementary file 6 [file Image1.tif]

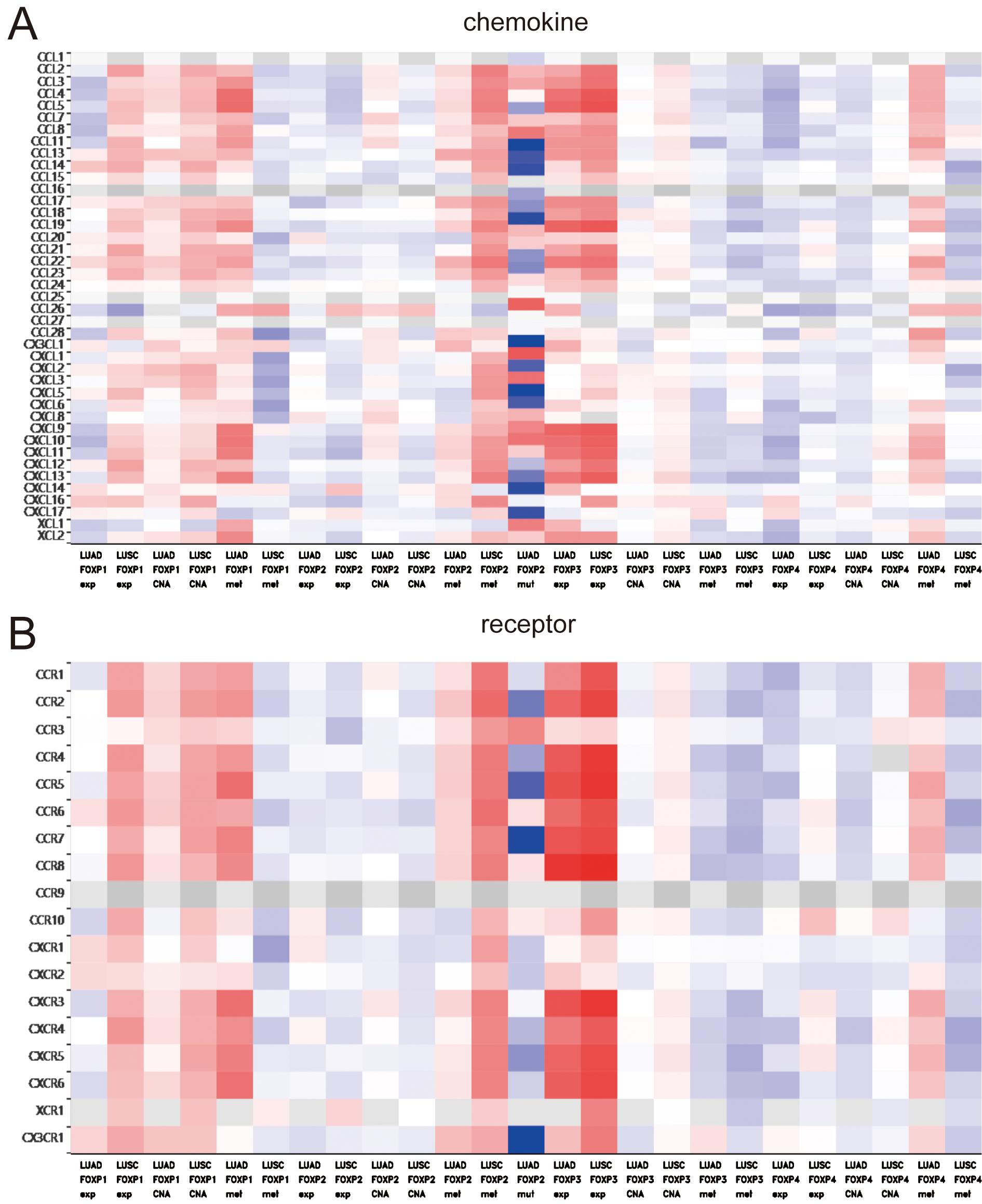

Supplement: Supplementary file 7 [file Image7.tif]

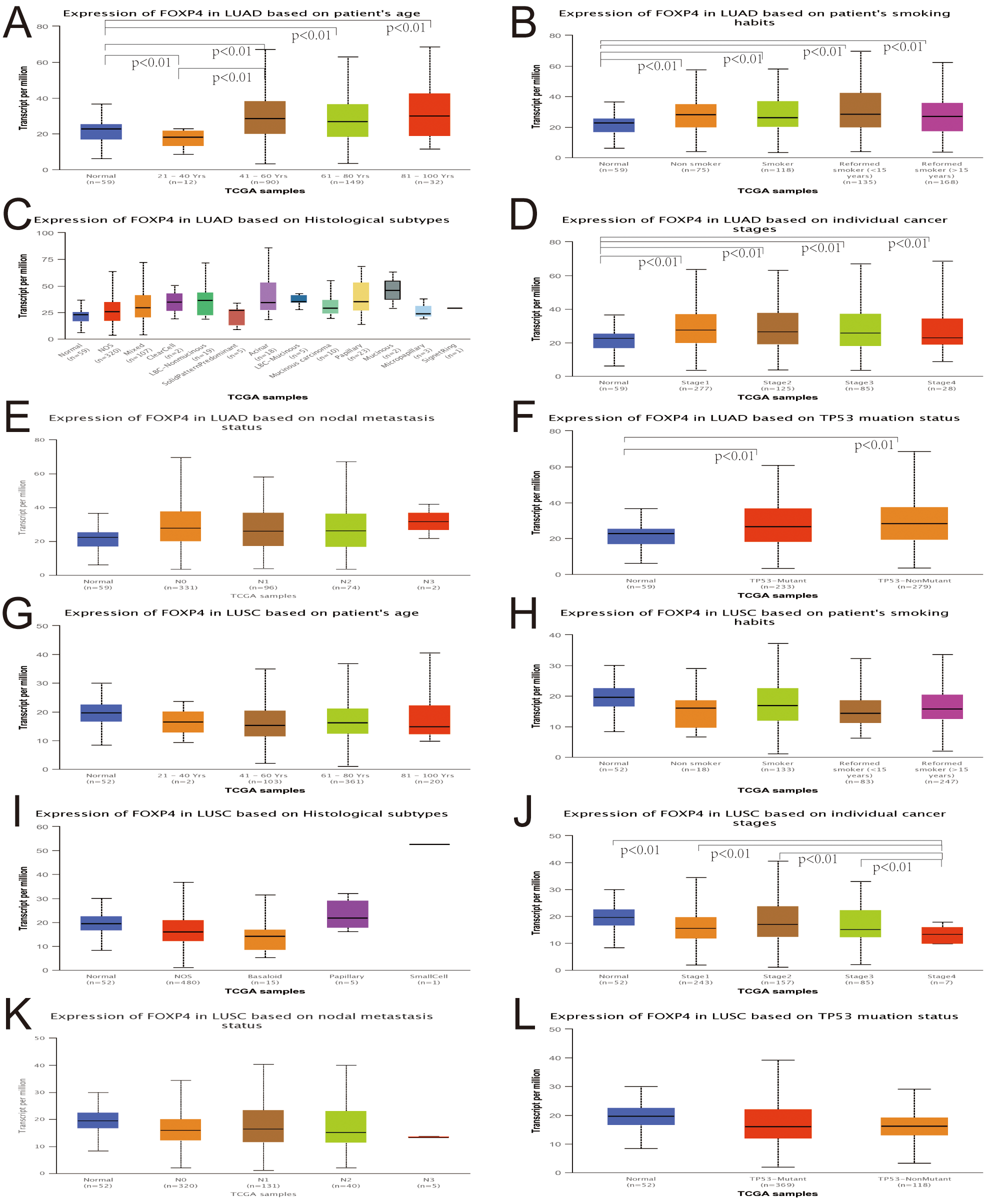

Supplement: Supplementary file 10 [file Image5.tif]
