# Supplementary material for: FOXP family DNA methylation correlates with immune infiltration and prognostic value in NSCLC
Source: Front Genet. 2022 Sep 9;13:937069. doi: 10.3389/fgene.2022.937069 (PMC9500381; doi:10.3389/fgene.2022.937069)
Supplement: Supplementary file 2 [file Table1.docx]

**Supplementary Table 1 Primer sequences of FOXP family and GAPDH used in RT-qPCR.**

| Targets | Primer sequences |
| --- | --- |
| FOXP1 | 160F GATTTGCTGTCAGCCATGAA |
|  | 160R GGTCACGTCTTACCCCTGAA |
| FOXP2 | 155F GAACACGCATTGGATGACCG |
|  | 155R TTGGGAGATGGTTTGGGCTC |
| FOXP3 | 90F CCTACCCACTGCTGGCAAAT |
|  | 90R CCTGGCAGTGCTTGAGGAA |
| FOXP4 | 129F GTGAGATGAGTCCCGCAGAG |
|  | 129R AGGCAGACTGTTTGCTGTCA |
| GAPDH | 138F GCACCGTCAAGGCTGAGAAC |
|  | 138R TGGTGAAGACGCCAGTGGA |

(Notes: RT-qPCR: reverse transcription quantitative polymerase chain reaction, FOXP1: Forkhead box P 1; FOXP2: Forkhead box P 2; FOXP3: Forkhead box P 3; FOXP4: Forkhead box P 4; GAPDH: glyceraldehyde 3-phosphate dehydrogenase;F: forward; R: reverse.)
