# Supplementary material for: FOXP family DNA methylation correlates with immune infiltration and prognostic value in NSCLC
Source: Front Genet. 2022 Sep 9;13:937069. doi: 10.3389/fgene.2022.937069 (PMC9500381; doi:10.3389/fgene.2022.937069)
Supplement: Supplementary file 8 [file Table2.docx]

**Supplementary Table 2 The sum of log-likelihood scores from all co-expression links (LLS score) is listed.**

| Gene | Rank | Co-expression gene Symbol | Co-expression gene Name | Score |
| --- | --- | --- | --- | --- |
| FOXP1 | 1 | PIK3IP1 | phosphoinositide-3-kinase interacting protein 1 | 6.808 |
|  | 2 | TGFBR2 | transforming growth factor beta receptor II | 5.518 |
|  | 3 | FOXO1 | forkhead box O1 | 4.112 |
|  | 4 | TIMP2 | TIMP metallopeptidase inhibitor 2 | 3.748 |
|  | 5 | CAMK4 | calcium/calmodulin-dependent protein kinase IV | 3.707 |
|  | 6 | TNS1 | tensin 1 | 3.543 |
|  | 7 | ZEB1 | zinc finger E-box binding homeobox 1 | 3.257 |
|  | 8 | PSIP1 | PC4 and SFRS1 interacting protein 1 | 2.9 |
|  | 9 | AEBP1 | AE binding protein 1 | 2.761 |
|  | 10 | MCOLN2 | mucolipin 2 | 2.755 |
|  | 11 | NFIB | nuclear factor I/B | 2.669 |
|  | 12 | BTG1 | B-cell translocation gene 1, anti-proliferative | 2.66 |
|  | 13 | RCAN3 | RCAN family member 3 | 2.656 |
|  | 14 | AFF3 | AF4/FMR2 family member 3 | 2.548 |
|  | 15 | AGR2 | anterior gradient 2, protein disulphide isomerase family member | 2.492 |
|  | 16 | EML6 | echinoderm microtubule associated protein like 6 | 2.462 |
|  | 17 | TBC1D9 | TBC1 domain family member 9 | 2.407 |
|  | 18 | BEX2 | brain expressed X-linked 2 | 2.304 |
|  | 19 | MAST4 | microtubule associated serine/threonine kinase family member 4 | 2.298 |
|  | 20 | TNFRSF10B | tumor necrosis factor receptor superfamily member 10b | 2.259 |
|  | 21 | THEM4 | thioesterase superfamily member 4 | 2.236 |
|  | 22 | OLFML1 | olfactomedin like 1 | 2.204 |
|  | 23 | NELL2 | neural EGFL like 2 | 2.182 |
|  | 24 | ADRB2 | adrenoceptor beta 2 | 2.18 |
|  | 25 | PTPRC | protein tyrosine phosphatase, receptor type, C | 2.175 |
|  | 26 | LEF1 | lymphoid enhancer-binding factor 1 | 2.154 |
|  | 27 | LRCH3 | leucine-rich repeats and calponin homology (CH) domain containing 3 | 2.136 |
|  | 28 | DOCK9 | dedicator of cytokinesis 9 | 2.127 |
|  | 29 | HECA | hdc homolog, cell cycle regulator | 2.113 |
|  | 30 | TCF7 | transcription factor 7 (T-cell specific, HMG-box) | 2.098 |
|  | 31 | IL6ST | interleukin 6 signal transducer | 2.088 |
|  | 32 | ZNRF3 | zinc and ring finger 3 | 2.069 |
|  | 33 | UGCG | UDP-glucose ceramide glucosyltransferase | 2.061 |
|  | 34 | LPP | LIM domain containing preferred translocation partner in lipoma | 2.06 |
|  | 35 | BACH2 | BTB and CNC homology 1, basic leucine zipper transcription factor 2 | 2.045 |
|  | 36 | COL9A3 | collagen, type IX, alpha 3 | 2.043 |
|  | 37 | BZW2 | basic leucine zipper and W2 domains 2 | 2.038 |
|  | 38 | TCF4 | transcription factor 4 | 2.038 |
|  | 39 | RET | ret proto-oncogene | 1.99 |
|  | 40 | AP1S3 | adaptor related protein complex 1 sigma 3 subunit | 1.979 |
|  | 41 | MLPH | melanophilin | 1.965 |
|  | 42 | PHIP | pleckstrin homology domain interacting protein | 1.962 |
|  | 43 | KLHL28 | kelch like family member 28 | 1.929 |
|  | 44 | USP47 | ubiquitin specific peptidase 47 | 1.895 |
|  | 45 | BCL2 | B-cell CLL/lymphoma 2 | 1.84 |
|  | 46 | POU6F1 | POU class 6 homeobox 1 | 1.829 |
|  | 47 | ZNF521 | zinc finger protein 521 | 1.82 |
|  | 48 | ZFP28 | ZFP28 zinc finger protein | 1.814 |
|  | 49 | SLC7A6 | solute carrier family 7 (amino acid transporter light chain, y+L system), member 6 | 1.804 |
|  | 50 | RAPGEF6 | Rap guanine nucleotide exchange factor 6 | 1.79 |
|  | 51 | IKZF1 | IKAROS family zinc finger 1 | 1.782 |
|  | 52 | RAB30 | RAB30, member RAS oncogene family | 1.775 |
|  | 53 | FYN | FYN proto-oncogene, Src family tyrosine kinase | 1.775 |
|  | 54 | SCNN1A | sodium channel, non voltage gated 1 alpha subunit | 1.766 |
|  | 55 | PRKD3 | protein kinase D3 | 1.754 |
|  | 56 | ITPR1 | inositol 1,4,5-trisphosphate receptor, type 1 | 1.754 |
|  | 57 | ZEB2 | zinc finger E-box binding homeobox 2 | 1.746 |
|  | 58 | APBB1 | amyloid beta (A4) precursor protein-binding, family B, member 1 (Fe65) | 1.73 |
|  | 59 | ADIRF | adipogenesis regulatory factor | 1.722 |
|  | 60 | TNRC6B | trinucleotide repeat containing 6B | 1.707 |
|  | 61 | SARAF | store-operated calcium entry-associated regulatory factor | 1.698 |
|  | 62 | PHF14 | PHD finger protein 14 | 1.694 |
|  | 63 | AGR3 | anterior gradient 3, protein disulphide isomerase family member | 1.686 |
|  | 64 | ATP7A | ATPase, Cu++ transporting, alpha polypeptide | 1.672 |
|  | 65 | KAT6A | K(lysine) acetyltransferase 6A | 1.654 |
|  | 66 | ZNF75A | zinc finger protein 75a | 1.614 |
|  | 67 | PLD4 | phospholipase D family member 4 | 1.596 |
|  | 68 | CNRIP1 | cannabinoid receptor interacting protein 1 | 1.594 |
|  | 69 | ZCCHC24 | zinc finger, CCHC domain containing 24 | 1.574 |
|  | 70 | SATB1 | SATB homeobox 1 | 1.561 |
|  | 71 | TXK | TXK tyrosine kinase | 1.557 |
|  | 72 | UBASH3B | ubiquitin associated and SH3 domain containing B | 1.556 |
|  | 73 | JAZF1 | JAZF zinc finger 1 | 1.549 |
|  | 74 | ZHX2 | zinc fingers and homeoboxes 2 | 1.516 |
|  | 75 | TBC1D4 | TBC1 domain family member 4 | 1.508 |
|  | 76 | ECM2 | extracellular matrix protein 2, female organ and adipocyte specific | 1.502 |
|  | 77 | GIMAP6 | GTPase, IMAP family member 6 | 1.491 |
|  | 78 | GRK5 | G protein-coupled receptor kinase 5 | 1.482 |
|  | 79 | TNFSF4 | tumor necrosis factor superfamily member 4 | 1.481 |
|  | 80 | SERPINB9 | serpin peptidase inhibitor, clade B (ovalbumin), member 9 | 1.479 |
|  | 81 | SYNE1 | spectrin repeat containing, nuclear envelope 1 | 1.479 |
|  | 82 | CD28 | CD28 molecule | 1.474 |
|  | 83 | PPP6R2 | protein phosphatase 6 regulatory subunit 2 | 1.436 |
|  | 84 | KANK2 | KN motif and ankyrin repeat domains 2 | 1.426 |
|  | 85 | GPRASP1 | G protein-coupled receptor associated sorting protein 1 | 1.424 |
|  | 86 | CHMP7 | charged multivesicular body protein 7 | 1.42 |
|  | 87 | JMJD1C | jumonji domain containing 1C | 1.408 |
|  | 88 | KIAA0226L | KIAA0226-like | 1.408 |
|  | 89 | TRPM4 | transient receptor potential cation channel, subfamily M, member 4 | 1.406 |
|  | 90 | KIAA1033 | KIAA1033 | 1.401 |
|  | 91 | CGNL1 | cingulin-like 1 | 1.39 |
|  | 92 | ZNF395 | zinc finger protein 395 | 1.387 |
|  | 93 | KLF9 | Kruppel-like factor 9 | 1.366 |
|  | 94 | CLEC2B | C-type lectin domain family 2 member B | 1.36 |
|  | 95 | FOXP2 | forkhead box P2 | 1.356 |
|  | 96 | FHL1 | four and a half LIM domains 1 | 1.343 |
|  | 97 | KIAA1324 | KIAA1324 | 1.338 |
|  | 98 | ADARB1 | adenosine deaminase, RNA-specific, B1 | 1.333 |
|  | 99 | NUCKS1 | nuclear casein kinase and cyclin-dependent kinase substrate 1 | 1.329 |
|  | 100 | DICER1 | dicer 1, ribonuclease type III | 1.319 |
|  | 101 | TMCC1 | transmembrane and coiled-coil domain family 1 | 1.317 |
|  | 102 | LDHB | lactate dehydrogenase B | 1.301 |
|  | 103 | FNBP4 | formin binding protein 4 | 1.296 |
|  | 104 | STX7 | syntaxin 7 | 1.28 |
|  | 105 | IGF1R | insulin like growth factor 1 receptor | 1.27 |
|  | 106 | EPC1 | enhancer of polycomb homolog 1 (Drosophila) | 1.253 |
|  | 107 | SMURF2 | SMAD specific E3 ubiquitin protein ligase 2 | 1.252 |
|  | 108 | SULF1 | sulfatase 1 | 1.246 |
|  | 109 | GGA1 | golgi-associated, gamma adaptin ear containing, ARF binding protein 1 | 1.242 |
|  | 110 | ID2 | inhibitor of DNA binding 2, dominant negative helix-loop-helix protein | 1.241 |
|  | 111 | EEF2K | eukaryotic elongation factor 2 kinase | 1.239 |
|  | 112 | MORC2 | MORC family CW-type zinc finger 2 | 1.209 |
|  | 113 | ITPKB | inositol-trisphosphate 3-kinase B | 1.203 |
|  | 114 | C1orf228 | chromosome 1 open reading frame 228 | 1.202 |
|  | 115 | PTPRN2 | protein tyrosine phosphatase, receptor type, N polypeptide 2 | 1.2 |
|  | 116 | CASC10 | cancer susceptibility candidate 10 | 1.2 |
|  | 117 | LAMA5 | laminin subunit alpha 5 | 1.195 |
|  | 118 | PRDX2 | peroxiredoxin 2 | 1.154 |
|  | 119 | ZNF101 | zinc finger protein 101 | 1.154 |
|  | 120 | CASK | calcium/calmodulin-dependent serine protein kinase (MAGUK family) | 1.139 |
|  | 121 | CASD1 | CAS1 domain containing 1 | 1.132 |
|  | 122 | ABLIM1 | actin binding LIM protein 1 | 1.13 |
|  | 123 | PLCL2 | phospholipase C like 2 | 1.129 |
|  | 124 | RRAS2 | related RAS viral (r-ras) oncogene homolog 2 | 1.126 |
|  | 125 | DGKA | diacylglycerol kinase alpha | 1.088 |
|  | 126 | ITK | IL2-inducible T-cell kinase | 1.085 |
|  | 127 | ERG | v-ets avian erythroblastosis virus E26 oncogene homolog | 1.081 |
|  | 128 | ZNF148 | zinc finger protein 148 | 1.056 |
|  | 129 | C11orf30 | EMSY, BRCA2-interacting transcriptional repressor | 1.056 |
|  | 130 | ATHL1 | ATH1, acid trehalase-like 1 (yeast) | 1.053 |
| FOXP2 | 1 | SLC4A4 | solute carrier family 4 (sodium bicarbonate cotransporter), member 4 | 4.016 |
|  | 2 | CXADR | coxsackie virus and adenovirus receptor | 3.892 |
|  | 3 | PDE1A | phosphodiesterase 1A | 3.699 |
|  | 4 | ERBB4 | erb-b2 receptor tyrosine kinase 4 | 3.307 |
|  | 5 | FOLH1 | folate hydrolase (prostate-specific membrane antigen) 1 | 3.097 |
|  | 6 | NAV3 | neuron navigator 3 | 3.056 |
|  | 7 | FIGN | fidgetin | 2.877 |
|  | 8 | CNTNAP3 | contactin associated protein-like 3 | 2.826 |
|  | 9 | TMEM241 | transmembrane protein 241 | 2.685 |
|  | 10 | ZNF521 | zinc finger protein 521 | 2.644 |
|  | 11 | ADGRL3 | adhesion G protein-coupled receptor L3 | 2.522 |
|  | 12 | GPM6A | glycoprotein M6A | 2.315 |
|  | 13 | COL3A1 | collagen, type III, alpha 1 | 2.161 |
|  | 14 | PAPPA | pregnancy-associated plasma protein A, pappalysin 1 | 2.147 |
|  | 15 | CSRNP3 | cysteine-serine-rich nuclear protein 3 | 2.144 |
|  | 16 | PCDH17 | protocadherin 17 | 2.064 |
|  | 17 | CYP2C9 | cytochrome P450 family 2 subfamily C member 9 | 1.936 |
|  | 18 | MYO1B | myosin IB | 1.93 |
|  | 19 | PTPRG | protein tyrosine phosphatase, receptor type, G | 1.922 |
|  | 20 | SYNPO2 | synaptopodin 2 | 1.921 |
|  | 21 | SCD5 | stearoyl-CoA desaturase 5 | 1.854 |
|  | 22 | PTN | pleiotrophin | 1.853 |
|  | 23 | DNAH7 | dynein, axonemal, heavy chain 7 | 1.845 |
|  | 24 | ADAD1 | adenosine deaminase domain containing 1 | 1.81 |
|  | 25 | SLC26A4 | solute carrier family 26 (anion exchanger), member 4 | 1.798 |
|  | 26 | SHANK2 | SH3 and multiple ankyrin repeat domains 2 | 1.779 |
|  | 27 | FLRT3 | fibronectin leucine rich transmembrane protein 3 | 1.752 |
|  | 28 | ATP6V0D2 | ATPase, H+ transporting, lysosomal 38kDa, V0 subunit d2 | 1.709 |
|  | 29 | SERPINB13 | serpin peptidase inhibitor, clade B (ovalbumin), member 13 | 1.645 |
|  | 30 | PBX1 | pre-B-cell leukemia homeobox 1 | 1.639 |
|  | 31 | BMPR1B | bone morphogenetic protein receptor type IB | 1.602 |
|  | 32 | ITGBL1 | integrin subunit beta like 1 | 1.601 |
|  | 33 | EDIL3 | EGF-like repeats and discoidin I-like domains 3 | 1.595 |
|  | 34 | CCDC13 | coiled-coil domain containing 13 | 1.552 |
|  | 35 | SH3GL3 | SH3-domain GRB2-like 3 | 1.551 |
|  | 36 | SLC22A15 | solute carrier family 22 member 15 | 1.544 |
|  | 37 | EFEMP1 | EGF containing fibulin-like extracellular matrix protein 1 | 1.525 |
|  | 38 | PQLC2L | PQ loop repeat containing 2-like | 1.506 |
|  | 39 | HTR2A | 5-hydroxytryptamine (serotonin) receptor 2A, G protein-coupled | 1.48 |
|  | 40 | FKBP10 | FK506 binding protein 10 | 1.458 |
|  | 41 | ACTN2 | actinin, alpha 2 | 1.44 |
|  | 42 | MMP16 | matrix metallopeptidase 16 | 1.44 |
|  | 43 | PSG6 | pregnancy specific beta-1-glycoprotein 6 | 1.43 |
|  | 44 | ASB10 | ankyrin repeat and SOCS box containing 10 | 1.418 |
|  | 45 | ADAMTS3 | ADAM metallopeptidase with thrombospondin type 1 motif 3 | 1.416 |
|  | 46 | MECOM | MDS1 and EVI1 complex locus | 1.408 |
|  | 47 | GDA | guanine deaminase | 1.364 |
|  | 48 | RUNX1T1 | runt-related transcription factor 1; translocated to, 1 (cyclin D-related) | 1.364 |
|  | 49 | FOXP1 | forkhead box P1 | 1.356 |
|  | 50 | RIMS2 | regulating synaptic membrane exocytosis 2 | 1.353 |
|  | 51 | SGCD | sarcoglycan delta | 1.342 |
|  | 52 | PTGS2 | prostaglandin-endoperoxide synthase 2 (prostaglandin G/H synthase and cyclooxygenase) | 1.335 |
|  | 53 | DLGAP1 | discs, large (Drosophila) homolog-associated protein 1 | 1.323 |
|  | 54 | YAP1 | Yes associated protein 1 | 1.313 |
|  | 55 | BCL2 | B-cell CLL/lymphoma 2 | 1.295 |
|  | 56 | CCDC40 | coiled-coil domain containing 40 | 1.292 |
|  | 57 | ADAM22 | ADAM metallopeptidase domain 22 | 1.287 |
|  | 58 | CES3 | carboxylesterase 3 | 1.273 |
|  | 59 | EEF2K | eukaryotic elongation factor 2 kinase | 1.239 |
|  | 60 | HMGA2 | high mobility group AT-hook 2 | 1.228 |
|  | 61 | ELAVL2 | ELAV like neuron-specific RNA binding protein 2 | 1.223 |
|  | 62 | ANKRD18A | ankyrin repeat domain 18A | 1.216 |
|  | 63 | GRIK2 | glutamate receptor, ionotropic, kainate 2 | 1.203 |
|  | 64 | NFIB | nuclear factor I/B | 1.201 |
|  | 65 | PDE1C | phosphodiesterase 1C | 1.148 |
|  | 66 | FGF7 | fibroblast growth factor 7 | 1.127 |
|  | 67 | HFE | hemochromatosis | 1.107 |
|  | 68 | SBSPON | somatomedin B and thrombospondin type 1 domain containing | 1.104 |
|  | 69 | FAM171B | family with sequence similarity 171 member B | 1.096 |
|  | 70 | ADAMTS20 | ADAM metallopeptidase with thrombospondin type 1 motif 20 | 1.096 |
|  | 71 | ADAM21 | ADAM metallopeptidase domain 21 | 1.096 |
|  | 72 | GALNT15 | polypeptide N-acetylgalactosaminyltransferase 15 | 1.093 |
|  | 73 | CYP19A1 | cytochrome P450 family 19 subfamily A member 1 | 1.084 |
|  | 74 | POTEM | POTE ankyrin domain family member M | 1.07 |
|  | 75 | PRKAA2 | protein kinase, AMP-activated, alpha 2 catalytic subunit | 1.064 |
|  | 76 | OPHN1 | oligophrenin 1 | 1.056 |
|  | 77 | WDR72 | WD repeat domain 72 | 1.032 |
|  | 78 | TRIM4 | tripartite motif containing 4 | 1.021 |
|  | 79 | ZNF479 | zinc finger protein 479 | 1.017 |
|  | 80 | ADGRG6 | adhesion G protein-coupled receptor G6 | 1.003 |
| FOXP3 | 1 | ZNF385A | zinc finger protein 385A | 1.4 |
|  | 2 | ADORA1 | adenosine A1 receptor | 1.273 |
|  | 3 | POFUT2 | protein O-fucosyltransferase 2 | 1.098 |
| FOXP4 | 1 | LEMD2 | LEM domain containing 2 | 2.38 |
|  | 2 | SF3A2 | splicing factor 3a subunit 2 | 2.285 |
|  | 3 | MAMLD1 | mastermind like domain containing 1 | 2.244 |
|  | 4 | ATXN2L | ataxin 2-like | 1.932 |
|  | 5 | EGFR | epidermal growth factor receptor | 1.863 |
|  | 6 | KRT16 | keratin 16, type I | 1.839 |
|  | 7 | CNOT3 | CCR4-NOT transcription complex subunit 3 | 1.72 |
|  | 8 | PIP5K1C | phosphatidylinositol-4-phosphate 5-kinase, type I, gamma | 1.708 |
|  | 9 | SCAMP4 | secretory carrier membrane protein 4 | 1.336 |
|  | 10 | KMT2D | lysine (K)-specific methyltransferase 2D | 1.248 |
|  | 11 | HCFC1 | host cell factor C1 | 1.181 |
|  | 12 | ABTB2 | ankyrin repeat and BTB (POZ) domain containing 2 | 1.165 |
