# Supplementary material for: FOXP family DNA methylation correlates with immune infiltration and prognostic value in NSCLC
Source: Front Genet. 2022 Sep 9;13:937069. doi: 10.3389/fgene.2022.937069 (PMC9500381; doi:10.3389/fgene.2022.937069)
Supplement: Supplementary file 9 [file Table3.docx]

**Supplementary Table 3 All median survival times, hazard ratios (HRs) with 95% confidence intervals, and the log-rank P values of Kaplan‒Meier curves are listed.**

| FOXP family | pathological types of lung cancer | Low expression cohort (months) | High expression cohort (months) | HRs(95% confidence intervals) | P value |
| --- | --- | --- | --- | --- | --- |
| FOXP1 | LUAD | 81.1 | 133.57 | 0.66(0.52-0.84) | 0.00075 |
|  | LUSC | 68 | 59.53 | 1.14(0.83-1.55) | 0.41 |
|  | NSCLC | 57 | 99.43 | 0.69(0.58-0.81) | 9e^-06^ |
| FOXP2 | LUAD | 108.97 | 90 | 1.31(1.03-1.67) | 0.027 |
|  | LUSC | 49.97 | 72 | 0.81(0.59-1.11) | 0.19 |
|  | NSCLC | 98.5 | 59 | 1.38(1.17-1.63) | 0.00012 |
| FOXP3 | LUAD | 112.67 | 90 | 1.37(1.09-1.73) | 0.0072 |
|  | LUSC | 62.47 | 45.6 | 1.2(0.95-1.53) | 0.13 |
|  | NSCLC | 78 | 61.21 | 1.25(1.1-1.41) | 0.00065 |
| FOXP4 | LUAD | 88.7 | 117.33 | 0.71(0.56-0.9) | 0.0053 |
|  | LUSC | 76.3 | 45.27 | 1.27(0.93-1.74) | 0.13 |
|  | NSCLC | 68.67 | 95.07 | 0.77(0.65-0.91) | 0.0017 |
